# Modeling Lymphoma Angiogenesis, Lymphangiogenesis, and Vessel Co-Option, and the Effects of Inhibition of Lymphoma–Vessel Interactions with an αCD20-EndoP125A Antibody Fusion Protein †

**DOI:** 10.3390/cells13221835

**Published:** 2024-11-06

**Authors:** Christian Elledge, Yu Zhang, Seung-Uon Shin, Hyun-Mi Cho, Sundaram Ramakrishnan, Ankita Sankar, Jennifer R. Chapman, Daniel Bilbao, Rathin Das, Hava Gil-Henn, Izidore S. Lossos, Joseph D. Rosenblatt

**Affiliations:** 1 Division of Hematology, University of Miami Miller School of Medicine, Miami, FL 33136, USA; 2 Department of Medicine, University of Miami Miller School of Medicine, Miami, FL 33136, USA; 3 Sheila & David Fuente Graduate Program in Cancer Biology, University of Miami Miller School of Medicine, Miami, FL 33136, USA; 4 Sylvester Comprehensive Cancer Center, University of Miami Miller School of Medicine, Miami, FL 33136, USA; 5 Department of Surgery, University of Miami Miller School of Medicine, Miami, FL 33136, USA; 6 Department of Pathology and Laboratory Medicine, University of Miami Miller School of Medicine, Miami, FL 33136, USA; 7 Synergys Biotherapeutics Inc., Alamo, CA 94595, USA; 8 The Azrieli Faculty of Medicine, Bar-Ilan University, Safed 13115, Israel

**Keywords:** lymphoma, mantle cell lymphoma, angiogenesis, lymphangiogenesis, vessel co-option, antibody fusion, endostatin

## Abstract

Lymphoma growth, progression, and dissemination require tumor cell interaction with supporting vessels and are facilitated through tumor-promoted angiogenesis, lymphangiogenesis, and/or lymphoma vessel co-option. Vessel co-option has been shown to be responsible for tumor initiation, metastasis, and resistance to anti-angiogenic treatment but is largely uncharacterized in the setting of lymphoma. We developed an in vitro model to study lymphoma–vessel interactions and found that mantle cell lymphoma (MCL) cells co-cultured on Matrigel with human umbilical vein (HUVEC) or human lymphatic (HLEC) endothelial cells migrate to and anneal with newly formed capillary-like (CLS) or lymphatic-like (LLS) structures, consistent with lymphoma–vessel co-option. To inhibit this interaction, we constructed an antibody fusion protein, αCD20-EndoP125A, linking mutant anti-angiogenic endostatin (EndoP125A) to an αCD20-IgG1-targeting antibody. αCD20-EndoP125A inhibited both CLS and LLS formation, as well as MCL migration and vessel co-option. Lymphoma vessel co-option requires cell migration, which is regulated by chemokine–chemokine receptor interactions. CXCL12 and its receptor, CXCR4, are highly expressed by both endothelial cells forming CLS and by MCL cells during vessel co-option. αCD20-EndoP125A suppressed expression of both CXCL12 and CXCR4, which were required to facilitate CLS assembly and vessel co-option. We also tested αCD20-EndoP125A effects in vivo using an aggressive murine B cell lymphoma model, 38c13-hCD20, which demonstrated rapid growth and dissemination to tumor-draining lymph nodes (TDLNs) and the spleen, lung, and brain. The pattern of lymphoma distribution and growth within the lung was consistent with vessel co-option. As predicted by our in vitro model, αCD20-EndoP125A treatment inhibited primary tumor growth, angiogenesis, and lymphangiogenesis, and markedly reduced the number of circulating tumor cells and lymphoma dissemination to TDLNs and the lungs, spleen, and brain. αCD20-EndoP125A inhibited lymphoma vessel co-option within the lung. Marked inhibition of MCL primary tumor growth and dissemination were also seen using an MCL xenograft model. The ability of αCD20-EndoP125A to inhibit angiogenesis, lymphangiogenesis, and lymphoma vessel co-option provides a novel therapeutic approach for inhibition of lymphoma progression and dissemination.

## 1. Introduction

Lymphoma progression requires the development of supporting vasculature and lymphatics. Interactions between malignant lymphoid cells and the tumor-supporting vessels are critical to growth, progression, and hematogenous spread [1]. Mantle cell lymphoma (MCL) is an aggressive non–Hodgkin’s lymphoma that frequently presents with advanced disease (Ann Arbor Stage III/IV) and extra-nodal involvement [2]. MCL and other lymphomas may spread locally or into extra-nodal sites through the lymphatics and/or circulation [3].

A variety of B cell lymphomas including Burkitt’s lymphoma, diffuse large B-cell lymphoma (DLBCL), and aggressive MCL have a propensity to invade into extranodal tissues, including the central nervous system, which drives morbidity and mortality [4]. Secondary CNS lymphoma preferentially affects the dura and leptomeninges and occurs in 4% to 11% of aggressive lymphoma patients [5]. Approximately 4% of MCL patients will manifest CNS involvement, and aggressive MCL such as the blastoid/pleomorphic variants have significantly higher rates of CNS involvement (reported in 28% of patients by the European MCL Network) [6]. Parenchymal involvement is more frequently seen in DLBCL whereas invasion into the leptomeninges is more common in aggressive MCL and Burkitt’s lymphoma [7]. Extranodal dissemination, and invasion into the CNS in particular, via the bloodstream or lymphatic system represent a major clinical challenge.

The formation of new tumor blood vessels (angiogenesis) and lymphatics (lymphangiogenesis) proceeds through the recruitment and differentiation of endothelial cells [8,9,10]. Vascular support for tumor growth and progression could also be provided by vessel co-option, i.e., malignant cell migration to and utilization of pre-existing normal vasculature [11]. Vessel co-option in solid tumors is reported to play a role in tumor initiation and metastasis and has been implicated in anti-angiogenic resistance [12,13,14]. Vessel co-option has been commonly described in solid tumors involving the lungs and brain for primary and metastatic tumors [15]. Vessel co-option within the lungs was observed in primary non-small-cell lung cancers [16] and in metastatic breast, colorectal, and renal cancers that were shown to be anti-angiogenic-resistant [17]. Co-option of pre-existing brain capillaries that preceded angiogenesis has been described in glioblastoma multiforme [18]. Vessel co-option was also identified in metastatic brain lesions originating from breast, melanoma, colorectal, and lung cancers [19]. Therefore, vessel co-option may support tumor initiation, metastasis, and resistance to treatment. However, in contrast to angiogenesis, the cellular and molecular mechanisms for vessel co-option are less well-characterized and the role of vessel co-option in lymphoma pathogenies is poorly understood. We therefore developed an in vitro assay for the association of lymphoma cells with pre-formed vasculature as a means of studying interactions involved in tumor vessel co-option, and the effects of anti-angiogenic agents.

Although the targeting of tumor-supporting vasculature has been applied in solid tumors, refs. [9,10] targeting lymphoma interactions with vasculature has been less effective in preventing lymphoma progression and dissemination [20]. For example, Bevacizumab, which targets VEGF-A, is approved for the treatment of several solid tumors [20]. However, in a SWOG trial of relapsed DLBCL and MCL, Bevacizumab had limited single-agent activity [21]. In a randomized phase II trial, Rituximab combined with Bevacizumab in relapsed follicular lymphoma demonstrated modestly improved progression-free survival but not overall survival [22]. The addition of bevacizumab to R-CHOP in a phase II DLBCL trial increased cardiac events without significantly improving efficacy [23]. More effective means of inhibiting lymphoma vascularization could augment existing anti-angiogenic approaches.

Monoclonal antibodies, directed against surface antigens prevalent on lymphoma cells, are commonly used alone [24] or in combination with other targeted anti-neoplastic agents to treat hematological malignancies [25,26,27]. CD20 is a membrane-bound glycosylated phosphoprotein expressed on B cells and has been extensively targeted by monoclonal antibodies such as Rituximab in lymphoma treatment [28,29]. We developed a novel antibody fusion protein, αCD20-EndoP125A, designed to target the lymphoma interactions with supporting vasculature. αCD20-EndoP125A links an αCD20-IgG1-targeting antibody to a mutant version of human endostatin, EndoP125A. Endostatin, the parent molecule of EndoP125A, is an endogenously produced 22KDa fragment of collagen XVIII with anti-angiogenic properties [30,31,32,33]. We have incorporated a modified EndoP125A into our fusion protein in a manner that preserves critical N-terminal Zn-binding amino acids and substitutes proline for alanine at position 125, which confers increased binding to endothelial cells and increased anti-angiogenic activity compared to parent endostatin [34].

Linking endostatin or EndoP125A to a targeting antibody markedly increases serum half-life and targeted tumor delivery, thereby enhancing anti-tumor efficacy [35]. Our prior studies of fused EndoP125A with an αHER2 antibody demonstrated superior anti-angiogenic and anti-tumor efficacy to αHER2 antibody alone, wild-type endostatin, or αHER2-fused to wild-type endostatin against HER2^+^ breast cancer xenografts [35,36]. αEGFR-EndoP125A, a fusion that targeted EGFR, was more effective than both EndoP125A and the parental anti-EGFR antibody cetuximab at inhibiting endothelial angiogenesis, triple-negative breast cancer (TNBC) vasculogenic mimicry, and breast cancer cell motility in vitro. αEGFR-EndoP125A inhibited TNBC growth and metastasis and improved overall survival in highly metastatic TNBC xenograft models compared to cetuximab-treated controls [37,38].

In this report, we investigated lymphoma cell interactions with angiogenic and lymphatic endothelial cells and developed a rapid assay and model for the study of lymphoma vessel co-option. Using our in vitro modeling system, we investigated the effects of αCD20-EndoP125A and based on results seen in vitro, we tested the efficacy of αCD20-EndoP125A in vivo. We describe the effects of αCD20-EndoP125A on angiogenesis, lymphangiogenesis, lymphoma motility, and lymphoma vessel co-option in vitro and in vivo, providing a novel approach to inhibiting lymphoma growth and dissemination.

## 2. Materials and Methods

### 2.1. Cell Lines and Mice

Human umbilical vein endothelial cells (HUVECs), human lymphatic endothelial cells (HLECs), Mino, and Jeko-1 cell cultures were purchased from ATCC (Manassas, VA, USA) and 38c13-huCD20 was provided to us by Dr. John Timmerman, UCLA. MCL cell lines Granta-519, HBL-2, L-128, and Z-138 were provided by Dr. Izidore Lossos, University of Miami. Lymphoma cell cultures were cultured in RPMI-1640/10% FBS and 100 U/mL penicillin–streptomycin (Gibco-BRL; Waltham, MA, USA). HUVEC cells were cultured in EGM-2 (Lonza; Walkersville, MD, USA). C3H/HeJ (Strain#000659) and NSG (Strain#005557) mice were purchased from Jackson Laboratories (Bar Harbor, MA, USA).

### 2.2. Construction of EndostatinP125A(EndoP125A), Fc-EndoP125A, and αCD20-EndoP125A

EndoP125A [35] and Fc-EndoP125A [37] were expressed and purified as previously described.

Using publicly available amino acid sequences of Rituximab (http://www.drugbank.ca/drugs/DB00073, accessed on 11 June 2015), coding sequences for anti-CD20 heavy and light chains were deduced using the Vector NTI program (V1.5.1) (GenScript, Piscataway, NJ, USA). Heavy and light chain variable region gene coding sequences were synthesized and subcloned into an αEGFR-IgG1-huEndostatin-P125A expression vector as previously described [37]. αCD20-EndoP125A heavy and light chain genes were constructed by using αEGFR-IgG1-huEndostatin-P125A heavy and light chain coding sequences in a SMART Expression pCT™ vector (Celltheon, Union City, CA, USA) as a template and replacing αEGFR heavy and light chain variable region coding sequences with synthesized αCD20 heavy and light chain variable region genes (GenScript, Piscataway, NJ, USA), respectively. SMART Expression pCT™ expression vectors encoding αCD20-EndoP125A heavy chain and kappa light chain genes were transfected into CHO cells. Stably transduced CHO cells expressing αCD20-EndoP125A were selected, and fusion protein was purified from culture supernatants using a Mab Select SuRe column (GE Healthcare Life Sciences; Marlborough, MA, USA) followed by SE-HPLC, which resulted in >95% purity as determined by Western blots using the anti-IgG and anti-endostatin sera and SEC chromatography.

### 2.3. Western Blot of αCD20-EndoP125A

Rituximab or αCD20-EndoP125A antibody proteins were either non-reduced to measure the full-length antibody or reduced with 10% 1.5M β-Mercaptoethanol sample buffer to measure the heavy and light chains of the antibody. First, 4–20% polyacrylamide gels were loaded with 5 or 10 µg of proteins and run for 1 h at 75 volts, then transferred to a nitrocellulose membrane. Membranes were blocked in 5% skim milk. To detect IgG1, IgG1-HRP was used at a dilution of 1:2500. To detect endostatin, anti-human biotinylated endostatin at a dilution of 1:2500 was used as the primary antibody, and Avidin-HRP at a dilution of 1:2000 was used as the secondary antibody. Blots were washed 3× with PBST and then incubated with HRP substrate for 5min. Images were captured on a LI-COR Odyssey XF imager.

### 2.4. Antibody Binding

Cells were blocked with goat serum (Invitrogen; Carlsbad, CA, USA, cat#50062Z) and Human TruStain FcX (Biolegend; San Diego, CA, USA, cat#422302) then incubated with equimolar Fc-EndoP125A, Rituximab, or αCD20-EndoP125A for 1 h. Samples were washed with PBS and stained with αhuIgG-FITC (Sigma-Aldrich; St. Louis, MO, USA, cat#F0132). Cells were resuspended in staining buffer (1× PBS: 0.5%BSA, 2mM EDTA, 0.09%SA) and flow cytometry was performed on an Attune NXT cytometer.

### 2.5. Vessel Formation

Endothelial tube formation assays were performed as previously reported [35]. For MCL–vessel interactions, HUVECs or HLECs labeled with calcein red (Invitrogen; Carlsbad, CA, USA, cat#C34851) and MCLs labeled with calcein green (Invitrogen; Carlsbad, CA, USA, cat#C34852) were plated in a 1:1 ratio. Cultures were treated as indicated and imaged at indicated times.

### 2.6. Cell Tracking

HUVEC or Jeko-1 cells were cultured on Matrigel in EGM-2 media and treated with indicated treatments. Cell cultures were incubated in IncuCyte at 37 °C for 24 h and images captured in 10–20 min intervals. Cell tracks were traced, and average distance traversed calculated using ImageJ software (Version 2.14.0/1.54f) [38].

### 2.7. Transwell Migration

Transwell assays of lymphoma cell migration to endothelial cells were set up in 24-well plates (details for experimental set-up are depicted in Figure 3C). HUVECs were cultured at 1.5 × 10^5^ cells/750 μL EBM-2 media in the bottom chamber in the absence of Matrigel to form a monolayer or on Matrigel to form CLS. Then, 5 μm porous membrane inserts were added. MCL cells were labeled with calcein green or red and pre-treated with indicated treatments for 1h. MCL cells were washed with PBS to remove unbound treatment and 2 × 10^5^ calcein-labeled MCL cells/200 μL of serum-free media were seeded into the top chamber. Cultures were then incubated for 6 h at 37 °C. The top chamber containing non-migrated cells was removed and photos of the bottom chamber were taken to capture lymphoma cells that had migrated down to the endothelial cells. Quantification of the number of MCL cells which migrated to the HUVEC monolayer or to CLS was performed by ImageJ cell count analysis.

### 2.8. Multi-Analyte Protein Expression Assay

Jeko-1, HUVEC, or Jeko-1:HUVEC cocultures were cultured in media or on Matrigel. Cell cultures were either left untreated or treated with equimolar concentrations of Fc-EndoP125A, Rituximab, or αCD20-EndoP125A and incubated for 6 h at 37 °C. Cell culture supernatant was collected and ProcartaPlex multi-analyte protein expression assays were performed according to protocol (Thermofisher; Waltham, MA, USA, cat#EPX800-10080-901, cat#EPX180-15806-901, cat#PPX-06). Flow cytometry was performed on a FlexMap 3D plate reader and the mean fluorescence readout was used to quantify individual analyte concentrations. Analysis and conversion of the MFI to specific protein concentrations (pg/mL) were performed on Thermofisher’s ProcartaPlex app.

### 2.9. CXCR4 Expression

Cells were cultured in media or on Matrigel and treated as indicated. Cells were recovered from Matrigel with Cell Recovery Solution (Corning Inc.; Corning, NY, USA, cat#354270), blocked with human TruStain FcX (Biolegend), and stained with CD31-FITC (Invitrogen; Carlsbad, CA, USA, cat#BMS137FI) and CXCR4-PE (BD Biosciences; Franklin Lakes, NJ, USA, cat#55150). Samples were resuspended in staining buffer and flow cytometry was performed on an Attune NXT cytometer (Gating Strategy Supplementary Figure S6).

### 2.10. Jeko-1-Luc-GFP In Vivo Experiments

Jeko-1 cells were induced to express GFP/luciferase. A total of 3 × 10^6^ Jeko-1-Luc-GFP^+^ cells suspended in 200 μL PBS containing 10% Matrigel were implanted subcutaneously into the right hind flank of NSG mice (female/6 week, Jackson Laboratories). Starting on d14 post-implantation, mice were intraperitoneally treated 2×/week with 200 μL PBS or PBS containing equimolar concentrations of Rituximab (136 μg/200 μL) or αCD20-EndoP125A (200 μg/200 μL injection). Tumor size was assessed using a digital caliper and luciferase expression was measured weekly beginning in week 2. Mice were euthanized at experiment termination (5–6 weeks) and the tumors, spleen, lungs, liver, and brain were collected. Tissues were prepared into single-cell solutions and flow cytometry for GFP^+^ Jeko-1 MCL cells was performed on an Attune NXT cytometer (details for GFP/Luciferase expression and experimental data can be found in Supplementary Figure S9).

### 2.11. 38c13-hCD20 In Vivo Experiments

A total of 5 × 10^3^ 38c13-hCD20 [39] cells suspended in 200 μL of PBS containing 10% Matrigel were subcutaneously implanted into C3H mice (female/6 week, Jackson Laboratories). Mice were intraperitoneally treated with equimolar Rituximab, Fc-EndoP125A, or αCD20-EndoP125A on d8, d11, d13, and d16. Tumor growth was assessed using a digital caliper. Mice were euthanized for distress, abnormal behavior, or at experiment termination on day 20. Immediately following euthanasia, the tumors, peripheral blood, tumor-draining lymph nodes, spleen, lung, and brain were collected. Tissues were either prepared into single-cell solutions for flow cytometry or formalin-fixed and embedded in paraffin blocks for IHC.

### 2.12. Analysis of Murine Tissues by Immunofluorescent Staining and Flow Cytometry

Single-cell suspensions were prepared by gently mashing tissues through 100 μm filters. Cell suspensions were then blocked with purified rat anti-mouse CD16/CD32 (BD Biosciences; Franklin Lakes, NJ, USA, cat#553142) and stained with antibodies for 30 min at 4 °C in the dark. Samples were washed with PBS, resuspended in 500 μL of staining buffer (1× PBS: 0.5%BSA, 2mM EDTA, 0.09%SA), and flow cytometry was performed on a Cytek Aurora Spectral Analyzer (antibody list and gating strategy Supplementary Figure S11).

### 2.13. Analysis of Murine Tissues by Immunohistochemistry

For IHC analysis, FFPE tissue blocks were sectioned at 5 µm and loaded onto an automated immunostainer (Leica BOND III or Leica BOND RX, Leica Biosystems Ltd., Newcastle, UK). All sections underwent dewaxing, rehydration, heat-induced antigen retrieval, and blocking. The detection antibodies used were anti-murine CD34 (Abcam, Cambridge, United Kingdom, ab81289, dilution 1:200) to stain blood vessels, anti-murine LyVE-1 (Abcam ab218535, dilution 1:5000) to stain lymphatics, and anti-human CD20 to stain lymphoma cells. DAB staining was performed with Bond-Mixed DAB. Refining and counterstaining was done with light Hematoxylin.

### 2.14. Statistical Analysis and Illustrations

One-way ANOVA and unpaired two-tailed Student’s *t*-tests were used to determine *p*-values. A *p*-value < 0.05 was considered statistically significant, and the indicated *p*-values are * < 0.05, ** < 0.01, *** < 0.001, and **** < 0.0001. One-way ANOVA and unpaired Student’s *t*-tests were performed using GraphPad Prism software version 9. ImageJ (Version 2.14.0/1.54f) was used to quantify CLS formation by tubule meshes (angiogenesis analyzer plugin) [40], MCL alignment, and colocalization with CLS by Pearson’s correlation coefficients (JACoP plugin), transwell migration cell counts, percentage of tumor vasculature, and tumor mean vessel density. Figures were prepared using PowerPoint (Version 16.54) and Adobe Illustrator (v28.6 (2024)), and schematic diagrams were drawn using BioRender.

## 3. Results

### 3.1. MCL Cells Migrate to and Align with Capillary and Lymphatic Vasculature

**Figure 1:**
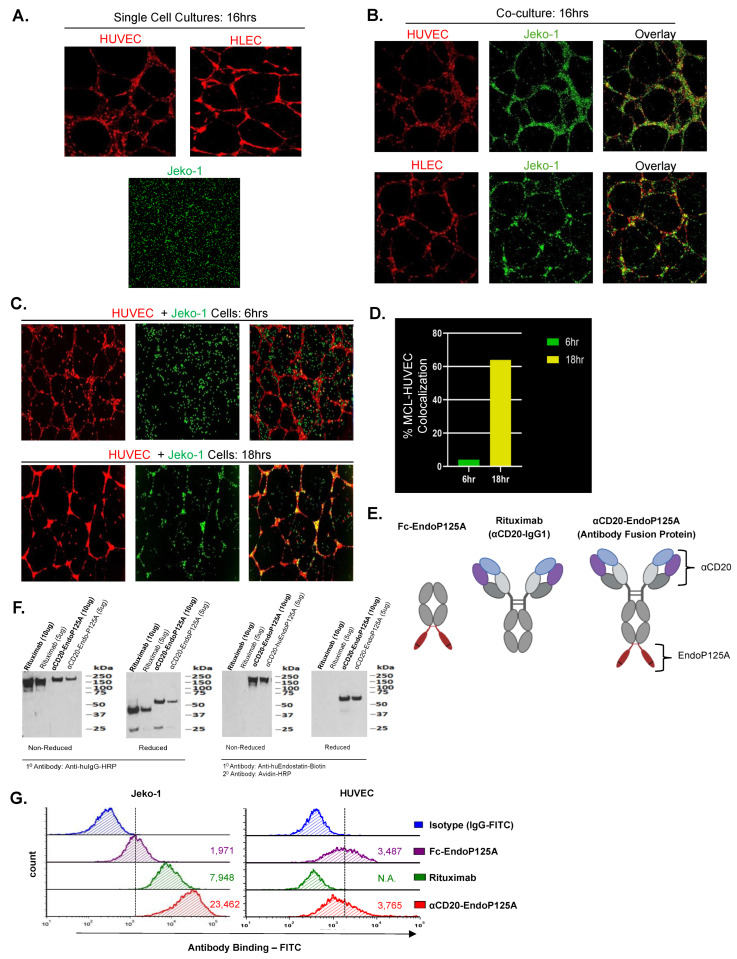
MCL migrates to and associates with lymphatic/vascular endothelial cells undergoing capillary-like structure (CLS) or lymphatic-like structure (LLS) assembly. Structure and binding of antibody fusion: αCD20-EndoP125A: (**A**–**C**) HUVECs or HLECs were labeled with calcein red and MCLs were labeled with calcein green. Cells were either cultured alone or co-cultured on Matrigel. Images were captured and displayed at 10× magnification. (**A**) Cultured-alone HUVECs form CLS, HLECs form LLS, and Jeko-1 MCL cells randomly disperse on Matrigel. (**B**) Jeko-1 cells co-cultured with HUVECs or with HLECs associated and aligned with CLS or LLS, respectively. (**C**) HUVECs were cultured on Matrigel for 6 h to form CLS. Jeko-1 cells were then seeded onto the pre-formed CLS. After an additional 12 h, Jeko-1 cells had migrated to and aligned with the pre-formed CLS. Pearson’s Coefficients were calculated using the ImageJ JACoP plugin to quantify Jeko-1:HUVEC colocalization: 6 h R = 0.069, 18 h R = 0.446. (**D**) Percentage of total Jeko-1 cells colocalized with HUVEC CLS at 6 h and 18 h. (**E**) Schematic rendering of antibodies and the antibody fusion protein: Fc-EndoP125A, which lacks variable targeting sequences, Rituximab, and the antibody fusion αCD20-EndoP125A, with αCD20 and EndoP125A domains indicated. The details are in the text. (**F**) Western blot confirmation of endostatinP125A bound to the heavy chain of the antibody. Antibody proteins were either non-reduced to measure the full-length antibody or reduced to measure the heavy and light chains of the antibody. To detect IgG1, IgG1-HRP was used. To detect endostatin, anti-human biotinylated was used as the primary antibody, and Avidin-HRP was used as the secondary antibody. (**G**) Flow cytometry of αCD20-EndoP125A binding to Jeko-1 and HUVEC cells compared to Rituximab and Fc-EndoP125A. Dotted line indicates gating, and the calculated MFI is indicated.

To investigate the interactions between MCLs and endothelial cells, we used a vascular assembly model in which endothelial cells are cultured on Matrigel. Both HUVECs and HLECs cultured on Matrigel assembled into networks of capillary-like (CLS) or lymphatic-like (LLS) structures, mimicking angiogenesis or lymphangiogenesis, respectively [41]. Jeko-1 MCL cells cultured on Matrigel randomly dispersed throughout the Matrigel (Figure 1A). However, when Jeko-1 cells were co-cultured with HUVECs or with HLECs undergoing vessel formation, they migrated to and annealed with CLS or with LLS (Figure 1B). Similar migration and association with CLS were seen for five additional MCL cell lines (Supplementary Figure S1).

### 3.2. Development of a Model for Lymphoma Vessel Co-Option

We next tested MCL cell migration to and alignment with pre-formed CLS. Following culture of HUVECs on Matrigel for 6 h, leading to CLS formation, Jeko-1 cells were added to cultures and initially randomly dispersed on the Matrigel. During 12 h in co-culture, the MCL cells progressively migrated to and aligned with pre-formed CLS (Pearson’s Coefficients: 6 h R = 0.069, 18 h R = 0.446) (Figure 1C). The percentage of MCL cells aligned with CLS was found to be 4% at 6 h and increased to 64% at 18 h (Figure 1D). This model system allowed for in vitro study of lymphoma–vessel interactions with pre-formed vasculature; a process consistent with lymphoma vessel co-option.

### 3.3. Structure, Function, and Binding of αCD20-EndoP125A

We hypothesized that disrupting angiogenesis, lymphangiogenesis, or lymphoma vessel co-option would inhibit lymphoma growth and spread. We constructed an antibody fusion protein, αCD20-EndoP125A, designed to inhibit lymphoma–vessel interactions. The heavy chain sequences of Rituximab, an αCD20-IgG1 monoclonal antibody, were fused to sequences for EndoP125A [42]. The fusion of EndoP125A to the αCD20-targeting antibody enabled local delivery of EndoP125A as a dimeric therapeutic payload. As a control for the effects of CD20 targeting, we used Fc-EndoP125A, which lacked αCD20-targeting sequences but incorporated EndoP125A fused to an IgG1 heavy chain (Figure 1E) [37]. αCD20-EndoP125A has a molecular weight (MW) of ~220kDa, while Rituximab, lacking the EndoP125A domain, has a MW of ~150 kDa (Figure 1F).

We compared the binding of αCD20-EndoP125A, Rituximab, and Fc-EndoP125A using flow cytometry. Rituximab demonstrated increased binding to Jeko-1 cells compared to Fc-EndoP125A. However, αCD20-EndoP125A had ~3–5-fold increased binding to Jeko-1 cells in comparison to Rituximab, indicating antibody fusion binding through both αCD20 and EndoP125A domains. In contrast, αCD20-EndoP125A and Fc-EndoP125A bound to HUVECs at similar levels, while Rituximab did not bind, indicating that binding to endothelial cells was mediated through the EndoP125A domain (Figure 1G).

Rituximab, Fc-EndoP125A, or αCD20-EndoP125A did not directly induce apoptosis in Jeko-1 cells. However, both αCD20-EndoP125A and Rituximab could mediate antibody-dependent cellular cytotoxicity (ADCC) in the presence of cytotoxic NK effector cells (Supplementary Figure S2A,B).

### 3.4. αCD20-EndoP125A Inhibits the Assembly of CLS and LLS and the Association of MCLs with CLS and LLS

**Figure 2:**
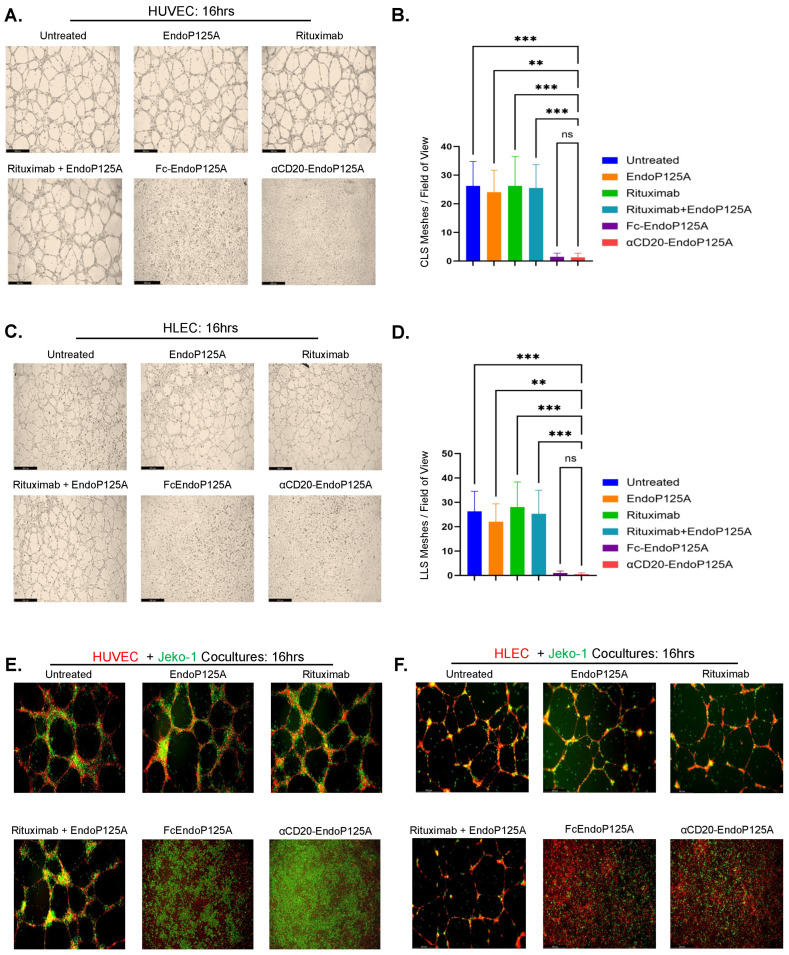
αCD20-EndoP125A inhibits the formation of CLS and LLS and consequently the association and alignment of MCL cells with CLS or LLS: (**A**–**F**) Cell cultures were cultured on Matrigel for 16h and either left untreated or treated with equimolar EndoP125A(2 μg/mL), Rituximab(6.8 μg/mL), EndoP125A + Rituximab, Fc-EndoP125A(3.5 μg/mL), or αCD20-EndoP125A(10 μg/mL). (**A**) HUVECs cultured on Matrigel for 16 h with indicated treatments. Images were captured and displayed at 5× magnification. (**B**) The number of tubule meshes formed in triplicate wells per treatment was calculated using the ImageJ angiogenesis analyzer and used to quantify the amount of CLS. Results are the mean number of meshes per 10× field of view ± Standard Deviation (SD). (**C**) HLECs cultured on Matrigel for 16 h with indicated treatments. Images were captured and displayed at 5× magnification (**D**) The number of tubule meshes formed in triplicate wells per treatment used to quantify the amount of LLS. Results are the mean number of meshes per 10× field of view ± SD. (**E**) HUVECs or (**F**) HLECs were labeled with calcein red, and Jeko-1 cells were labeled with calcein green. Cells were co-cultured on Matrigel for 16h with indicated treatments. Images were captured and displayed at 10× magnification Pearson’s Coefficients calculated to quantify MCL colocalization with CLS (Untreated R = 0.353, EndoP125A R = 0.387, Rituximab R = 0.438, EndoP125A + Rituximab R = 0.422, Fc-EndoP125A R = 0.079, and αCD20-EndoP125A R = 0.129) and MCL colocalization with LLS (Untreated R = 0.375, EndoP125A R = 0.495, Rituximab R = 0.282, EndoP125A + Rituximab R = 0.293, Fc-EndoP125A R = −0.018, and αCD20-EndoP125A R = −0.021). *p*-value < 0.05 was considered statistically significant or marked ns for not significant. Indicated *p*-values are * < 0.05, ** < 0.01, *** < 0.001, and **** < 0.0001.

We tested the effects of αCD20-EndoP125A on the assembly of CLS and LLS. Dimerized Endostatin or EndostatinP125A incorporated into an antibody–endostatin fusion has been reported to inhibit CLS formation more efficiently than Endostatin delivered as a monomer [35,43]. Both Fc-EndoP125A and αCD20-EndoP125A, which deliver EndoP125A as a dimer, inhibited CLS formation, whereas Rituximab, EndoP125A, or their combination did not inhibit CLS formation, as measured by tubule mesh formation (Figure 2A,B). αCD20-EndoP125A also demonstrated significantly greater inhibition of CLS formation than Bevacizumab (Supplementary Figure S3).

Next, we assayed the effects of αCD20-EndoP125A on the assembly of lymphatics in vitro. Similar to HUVECs, HLEC assembly into LLS was inhibited by αCD20-EndoP125A or Fc-EndoP125A treatment but not by EndoP125A, Rituximab, or their combination (Figure 2C,D).

Furthermore, we tested the effects of αCD20-EndoP125A on MCL:HUVEC and MCL:HLEC co-cultures. αCD20-EndoP125A prevented CLS and LLS formation and, consequently, the alignment of MCL cells. Decreased colocalization of MCL with HUVEC or HLEC cells is represented by a decrease in Pearson’s correlation coefficients (Figure 2E,F).

### 3.5. αCD20-EndoP125A Reduces HUVEC and MCL Motility, Migration, and Invasion

**Figure 3:**
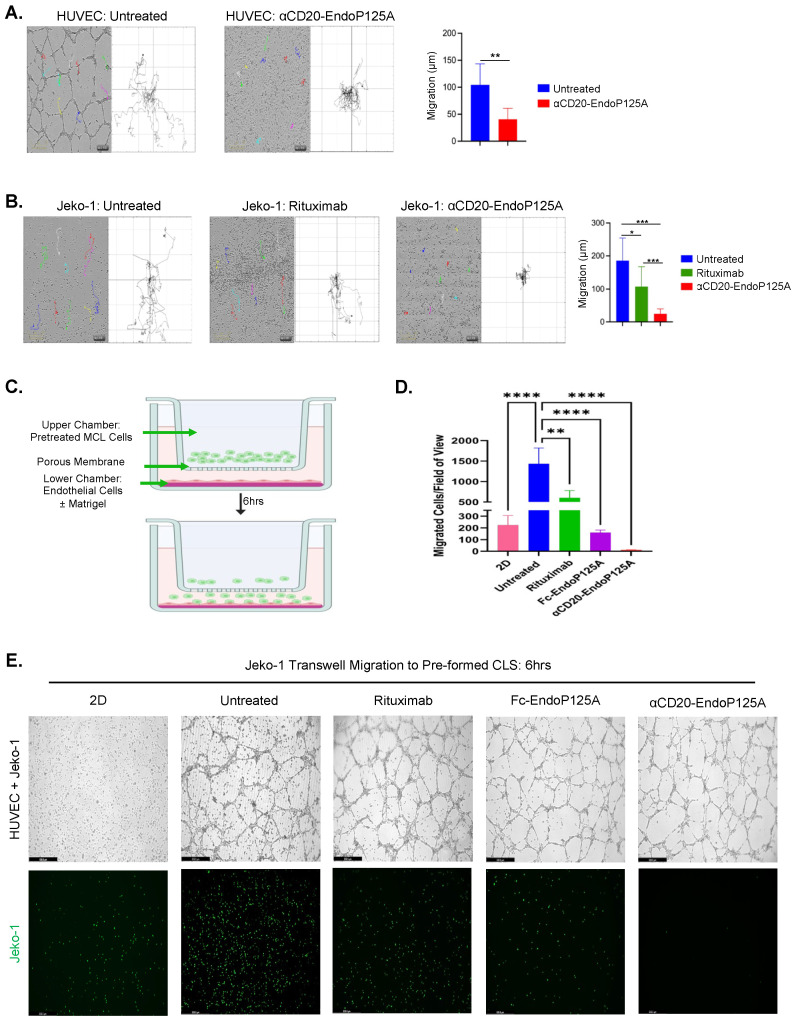
αCD20-EndoP125A significantly reduces MCL and endothelial cell motility and migration: (**A**) HUVEC or (**B**) Jeko-1 cells were cultured for 16h on Matrigel and were either untreated or treated with equimolar Rituximab (6.8 μg/mL) or αCD20-EndoP125A(10 μg/mL). Cells were randomly selected, and individual cell movements were tracked and plotted. Images displayed are 10× magnification and captured at the 16hr time point. The migration track of each individual cell is assigned a unique color. Corresponding spider plots display individual cell tracks starting at the plot’s origin and depict migration away from the point of origin. Distances traveled were quantified, averaged, and graphed. Results are the mean cell migration ± SD of 10 randomly chosen cells/condition. (**C**) Schematic rendering of transwell migration assay. In the lower chamber of a 24-well plate, 1.5 × 10^5^ HUVEC cells were cultured in Media alone to grow as a monolayer (labelled 2D) or on Matrigel to form CLS. Jeko-1 MCL cells were labeled with calcein green and pre-treated for 1hr with equimolar Rituximab (6.8 μg/mL), Fc-EndoP125A (3.5 μg/mL), or αCD20-EndoP125A (10 μg/mL). MCL cells were washed, 2.0 × 10^5^ cells were seeded in the top chamber, and transwells were incubated at 37 °C for 6 h. (**D**) Migration of green, fluorescent Jeko-1 cells to the bottom chamber of 4 wells per treatment was quantified by ImageJ software. Results are the mean number of migrated cells ± SD. (**E**) Representative images of the bottom chamber of transwell migration assays. Images were captured and displayed at 10× magnification. Top panel: phase contrast images depicting the endothelial monolayer or CLS, and Jeko-1 cells that migrated to the bottom chamber. Bottom panel: fluorescent images of the bottom chamber depicting green, fluorescent Jeko-1 cells that migrated to endothelial cells. *p*-value < 0.05 was considered statistically significant or marked ns for not significant. Indicated *p*-values are * < 0.05, ** < 0.01, *** < 0.001, and **** < 0.0001.

We hypothesized that failure to form CLS or LLS could be attributed to reduced endothelial cell motility. To test cell motility, HUVECs were cultured on Matrigel and either left untreated or treated with αCD20-EndoP125A. Time-lapse photography was used to track cell movements. Cells were chosen at random and ImageJ analysis was used to calculate the cumulative distance traveled by individually selected cells. αCD20-EndoP125A treatment significantly reduced HUVEC directional migration, which is necessary for CLS formation (~62% reduction, *p* < 0.001) (Figure 3A).

MCL cells spontaneously and randomly migrate throughout Matrigel. To test the effects on random migration, Jeko-1 MCL cells were cultured on Matrigel and were either untreated or treated with equimolar Rituximab or αCD20-EndoP125A. Treatment with Rituximab modestly reduced random migration of MCL cells (~42% reduction, *p* < 0.01). However, αCD20-EndoP125A treatment further inhibited migration and rendered MCL cells nearly immobile (~86% reduction, *p* < 0.001) (Figure 3B). We also tested MCL migration and invasion through a 10% Matrigel layer using serum-containing media as a stimulant (Supplementary Figure S4A,C). We found that αCD20-EndoP125A significantly reduced both directed migration and invasion (Supplementary Figure S4B,D).

To investigate MCL migration toward HUVEC cells, we utilized two-chamber transwells with HUVECs cultured in the bottom chamber to stimulate MCL migration. HUVECs were either cultured in the absence of Matrigel to form a monolayer (labeled as 2D) or on Matrigel to form CLS. MCL cells were seeded into the top chamber and after a period of 6h, the number of MCL cells that migrated to HUVECs was quantified (Figure 3C). MCL migration significantly increased to HUVEC CLS in Matrigel in comparison to 2D HUVEC monolayers (5-fold increase, *p* < 0.001). This demonstrated that lymphoma cells preferentially migrate toward pre-formed CLS and provided evidence of lymphoma vessel co-option in vitro. Furthermore, we assayed the effect of αCD20-EndoP125A on MCL vessel co-option. MCL cells were pre-treated for 1hr with either Rituximab, Fc-EndoP125A, or αCD20-EndoP125A prior to seeding into the top chamber. MCL migration was moderately reduced by Rituximab (~30% reduction, *p* < 0.01), further reduced by Fc-EndoP125A (~80% reduction, *p* < 0.0001), and nearly completely ablated by αCD20-EndoP125A (>98% reduction, *p*-value < 0.0001) (Figure 3D,E). Of note, following MCL migration to the bottom chamber, near complete alignment of MCL with CLS was seen at 10h (Supplementary Figure S4E).

### 3.6. αCD20-Endo-P125A Reduces Expression of CXCL12 and the Corresponding Chemokine Receptor CXCR4

Lymphoid cells are motile cells that traverse the lymphatics and circulation. Trafficking is tightly regulated by chemokines that locally activate lymphocytes in response to chemokine gradients [44]. Differential expression of chemokines and corresponding chemokine receptors results in selective leukocyte recruitment [45]. Chemokine interactions with chemokine receptors support lymphocyte adherence to the endothelium and transendothelial migration for intravasation and extravasation. Dysregulated chemokine and chemokine receptor expression is implicated in a variety of human diseases, including cancer [46,47].

We hypothesized that chemokine–chemokine receptor interactions facilitate CLS formation as well as MCL vessel co-option. We tested for changes in the expression of 98 different chemokines and angiogenic proteins (Supplemental Table S2) using multiplex immunoassays to simultaneously quantify analytes of interest.

**Figure 4:**
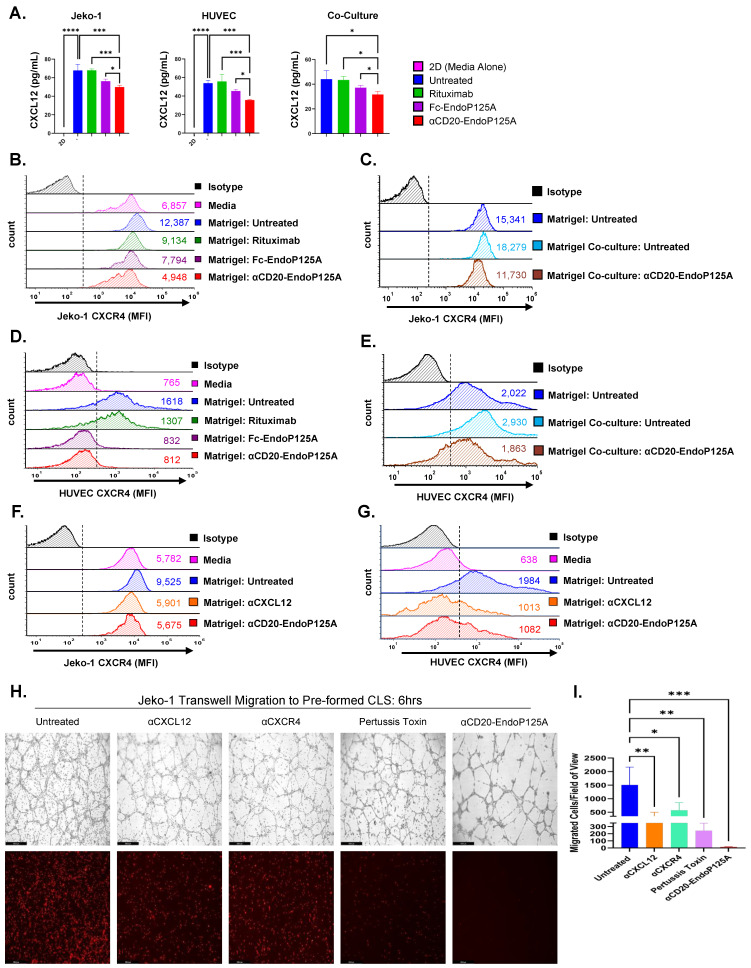
αCD20-EndoP125A reduces the expression of CXCL12 and CXCR4, which are critical for CLS formation and MCL vessel co-option: (**A**) Jeko-1, HUVEC, or Jeko-1:HUVEC cocultures were cultured in media alone (labeled 2D) or on Matrigel for 6h. Cultures were left untreated or treated with equimolar Rituximab (6.8 µg/mL), Fc-EndoP125A (3.5 µg/mL), or αCD20-EndoP125A (10 µg/mL). Supernatant was collected from triplicate wells per condition and ProcartaPlex Multi-Analyte Protein assays were performed to quantify analyte concentrations. Results are CXCL12 concentrations for indicated cultures and treatments ± SD (**B**–**G**) Jeko-1, HUVEC, or Jeko-1:HUVEC co-cultures were cultured in media alone or on Matrigel and left untreated or treated with Rituximab (6.8 µg/mL), Fc-EndoP125A (3.5 µg/mL), αCXCL12 (10 µg/mL), or αCD20-EndoP125A (10 µg/mL). Cells were recovered, stained, and flow cytometry was performed for CXCR4 expression. Dotted line indicates gating for CXCR4^+^ cells and calculated MFI is displayed. (**F**,**G**) Effects on CXCR4 expression through CXCL12 inhibition with an αCXCL12 antibody compared to αCD20-EndoP125A. (**H**) Transwell assays were performed as indicated previously (Figure 3C) to measure the effect of chemokine inhibitors compared to αCD20-EndoP125A. Treatment concentrations used were αCXCL12 (10 µg/mL), αCXCR4 (10 µg/mL), Pertussis Toxin (200 ng/mL), and αCD20-EndoP125A (10 µg/mL). Images were captured and displayed at 10× magnification. Top panel: phase contrast images of the bottom chamber depicting HUVEC CLS and Jeko-1 cells that migrated to the bottom chamber. Bottom panel: fluorescent images of the bottom chamber, depicting red, fluorescent Jeko-1 cells that migrated to endothelial cells. (**I**) Cell migration quantified by counting the number of red, fluorescent cells in the bottom chamber of 4 wells per treatment. Results are the mean number of migrated cells ± SD. *P*-value < 0.05 was considered statistically significant or marked ns for not significant. Indicated *p*-values are * < 0.05, ** < 0.01, *** < 0.001, and **** < 0.0001.

CXCL12 was the most highly expressed chemokine in the supernatant of Jeko-1, HUVEC, and Jeko-1:HUVEC cocultures on Matrigel and was significantly downregulated in cultures treated with αCD20-EndoP125A (Figure 4A and Figure S5A,B).

CXCL12 was not detected in HUVEC cultures in the absence of Matrigel (labeled 2D) but was significantly upregulated when HUVECs formed CLS on Matrigel. αCD20-EndoP125A treatment of HUVECs abrogated CLS formation and significantly decreased CXCL12 levels in comparison to Rituximab- or Fc-EndoP125A-treated cultures (Figure 4A).

CXCL12 was also not detectable in the supernatant of Jeko-1 cultures in media alone (labeled 2D), but expression significantly increased in Jeko-1 cultures on Matrigel. αCD20-EndoP125A markedly decreased the expression of CXCL12 compared to Rituximab or Fc-EndoP125A in both Jeko-1 cultures and in Jeko-1:HUVEC co-cultures (Figure 4A).

The CXCL12–CXCR4 axis is known to stimulate solid tumor vascularization through the recruitment of CXCR4^+^ proangiogenic cells to neoangiogenic niches, [48] and has been implicated in lymphoma progression, migration, and chemoresistance [48,49,50]. Flow cytometry for chemokine receptors demonstrated that CXCR4, the receptor for CXCL12, was highly expressed on Jeko-1 cells (Supplementary Figure S5C). CXCR4 expression on Jeko-1 cells also exceeded normal peripheral blood B cell expression (Supplementary Figure S5D). A query of the DepMap and GENT2 databases confirmed high CXCR4 mRNA expression in MCL cell lines and in MCL patient samples (Supplementary Figure S5E).

Jeko-1 CXCR4 expression increased following culture on Matrigel and further increased when Jeko-1 cells were co-cultured with HUVECs. Upregulation of Jeko-1 CXCR4 expression was suppressed with αCD20-EndoP125A (Figure 4B,C). Although baseline HUVEC CXCR4 expression was significantly lower than Jeko-1 cells, HUVEC CXCR4 was also markedly upregulated on Matrigel and further augmented when HUVECs were co-cultured with Jeko-1 cells. The increase in HUVEC CXCR4 expression was suppressed by αCD20-EndoP125A (Figure 4D,E). The increased CXCR4 expression observed on both Jeko-1 and HUVECs might lead to more bound and/or internalized CXCL12 and reduction of CXCL12 levels in the supernatant of HUVEC:Jeko-1 co-cultures. Treatment with an αCXCL12-neutralizing antibody reduced CXCR4 expression on HUVEC and Jeko-1 cells cultured on Matrigel to levels similar to αCD20-EndoP125A treatment (Figure 4F,G). In summary, the induction of CXCL12 and CXCR4 expression by HUVECs during CLS formation, or by Jeko-1 MCL during migration and vessel co-option, was suppressed by αCD20-EndoP125A.

### 3.7. αCXCL12- and αCXCR4-Neutralizing Antibodies Reduce CLS Formation and MCL Vessel Co-Option

We tested the effects on CLS formation by using chemokine-neutralizing antibodies specific to CXCL12 and CXCR4 and the non-specific inhibitor pertussis toxin, which targets all GPCR chemokine receptors [51]. HUVEC CLS network formation was reduced by αCXCL12, αCXCR4, and pertussis toxin, as measured by completed tubule mesh formation (Supplementary Figure S7).

We next assayed the effects of chemokine-neutralizing antibodies and pertussis toxin on MCL vessel co-option. In transwell assays, as previously described, Jeko-1 cells seeded in the top chamber were either untreated or pretreated with αCXCL12, αCXCR4, pertussis toxin, or αCD20-EndoP125A. αCXCL12 decreased Jeko-1 migration to pre-formed CLS by ~75% (*p* < 0.01), αCXCR4 by ~60% (*p* < 0.05), and pertussis toxin by ~80% (*p* < 0.01); however, αCD20-EndoP125A completely abrogated migration with a ~98% reduction (*p* < 0.001) (Figure 4H,I). Our data showed that (1) αCD20-EndoP125A reduces CXCL12 and CXCR4 expression and (2) the CXCL12–CXCR4 axis is critical for both vessel formation and MCL vessel co-option (Supplementary Figure S8).

### 3.8. αCD20-EndoP125A Inhibits Lymphoma Tumor Growth, Angiogenesis, Lymphangiogenesis, and Dissemination In Vivo

We initially investigated the effects of αCD20-EndoP125A in vivo using Jeko-1-Luc-GFP as a human MCL xenograft model. Both Rituximab and αCD20-EndoP125A significantly inhibited Jeko-1-Luc-GFP primary tumor growth and metastasis to the spleen, lung, liver, and brain (Supplementary Figure S9), which precluded an effective comparison of efficacy between Rituximab and αCD20-EndoP125A. We therefore chose to investigate an alternative model, 38C13-hCD20, an aggressive murine B cell lymphoma engineered to express human CD20 and shown to be Rituximab-resistant [52,53]. Similar to human MCLs, 38c13-hCD20 cells migrate to and align with CLS formed by murine C166 endothelial cells. αCD20-EndoP125A inhibits murine CLS formation and consequently the alignment of 38c13-hCD20 cells with CLS (Supplementary Figure S10).

**Figure 5:**
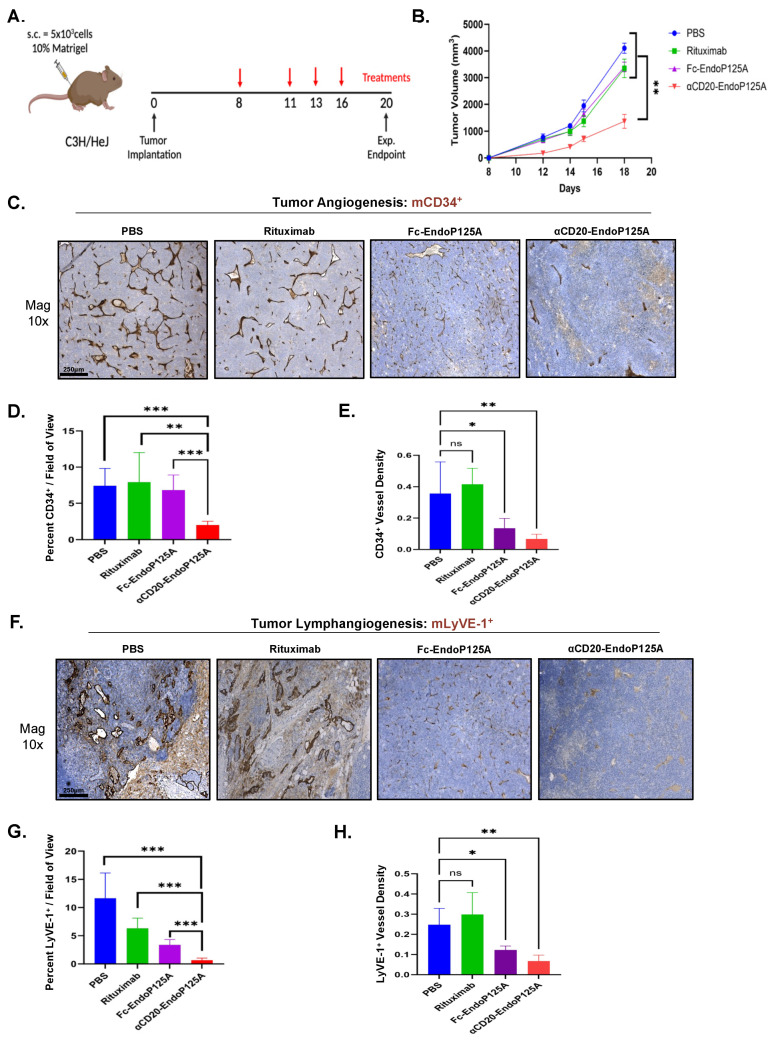
αCD20-EndoP125A reduces tumor growth, angiogenesis, and lymphangiogenesis: (**A**) Schematic rendering of the 38c13-hCD20 in vivo experiment. In total, 5 × 10^3^ 38c13-hCD20 cells suspended in 200 μL PBS containing 10% Matrigel were implanted s.c. in the right hind flank of C3H mice. A total of 10 mice/group were treated with 200 μL of PBS, or PBS containing equimolar Rituximab (136 μg/200 μL), Fc-EndoP125A (68 μg/200 μL), or αCD20-EndoP125A (200 μg/200 μL) on days 8, 11, 13, and 16. Mice were sacrificed on day 20 and tumors, peripheral blood, and organs were collected. (**B**) Mean tumor volume ± SEM of 10 mice/treatment group. (**C**) Representative images of tumor angiogenesis by IHC for mCD34. Quantification of tumor angiogenesis measured by (**D**) Percent mCD34^+^ per five 25× fields of view and by (**E**) Mean Vessel Density (MVD) per five 25× fields of view calculated by ImageJ software. (**F**) Representative images of tumor lymphangiogenesis by IHC staining tumors for mLyVE-1. Quantification of tumor lymphangiogenesis measured by (**G**) Percent mLyVE-1^+^ per five 25× fields of view of and by (**H**) MVD per five 25× fields of view calculated by ImageJ software. *p*-value < 0.05 was considered statistically significant or marked ns for not significant. Indicated *p*-values are * < 0.05, ** < 0.01, *** < 0.001, and **** < 0.0001.

To investigate the therapeutic efficacy of αCD20-EndoP125A in comparison to Rituximab in vivo, we tested the effects on lymphoma growth, angiogenesis, lymphangiogenesis, and dissemination. Mice were subcutaneously implanted with 38c13-hCD20 cells and treated with PBS or equimolar Rituximab, Fc-EndoP125A, or αCD20-EndoP125A on days 8, 11, 13, and 16 (Figure 5A). αCD20-EndoP125A treatment demonstrated significantly reduced primary tumor growth compared to all other treatments including Rituximab (Figure 5B). On d20, the mice were sacrificed, and tumors/organs were excised. Primary tumors from αCD20-EndoP125A-treated mice were both smaller and appeared less vascular (Supplementary Figure S12A). To measure tumor vascularity, we performed IHC using anti-murine CD34 to stain blood vessels and anti-murine LyVE-1 to stain lymphatic vessels (Figure 5C,F). Mice treated with αCD20-EndoP125A demonstrated a significant reduction in the total amount of both CD34^+^ and LyVE-1^+^ tumor vessels compared to all other treatments (Figure 5D,G). Fc-EndoP125A treatment did not decrease the total amount of tumor vasculature, but mice exhibited smaller tumor vessels and markedly decreased CD34^+^ and LyVE-1^+^ tumor vessel density compared to PBS- and Rituximab-treated mice (Figure 5E,H).

**Figure 6:**
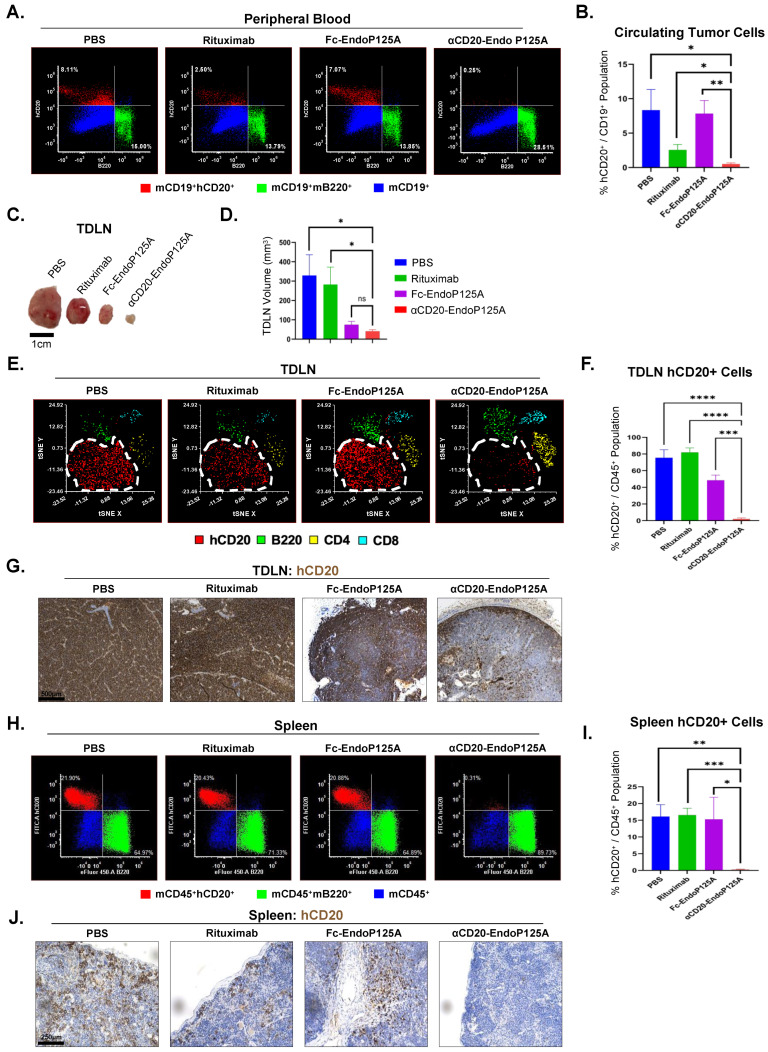
αCD20-EndoP125A reduces circulatory tumor cells (CTCs) and dissemination to tumor-draining lymph nodes (TDLN) and the spleen: (**A**) Representative flow cytometry of peripheral blood (PB) gated on mCD19^+^ cells depicting hCD20^+^ circulating tumor cells (CTCs, red) and mB220^+^ normal murine B cells (green). (**B**) Flow cytometry quantification of percentage of 38c13-hCD20 cells within PB. Results are the mean percentage of hCD20^+^ cells within the CD19^+^ population ± SD of 5 PB/treatment group. (**C**) Representative images of TDLN. (**D**) TDLN volume ± SD of 5 TDLN/treatment group. (**E**) tSNE plots indicating disseminated hCD20^+^ lymphoma cells (red), and normal mouse immune cells: mB220^+^ B cells (green), mCD4^+^ T cells (yellow), and mCD8^+^ T cells (blue) within TDLN. (**F**) Flow cytometry quantification of infiltrating 38c13-hCD20 cells within the TDLN. Results are the mean percentage of hCD20^+^ cells within the CD45^+^ population ± SD of 5 TDLN/treatment group. (**G**) Representative TDLN IHC for hCD20^+^ cells (brown) confirming αCD20-EndoP125A reduction of 38c13-hCD20 lymphoma infiltration. (**H**) Representative flow cytometry of the spleen depicting hCD20^+^ cells (red) and B220 cells (green) within the CD45^+^ cell population (blue). (**I**) Flow cytometry quantification of percentage of infiltrating 38c13-hCD20 lymphoma cells within the spleen. Results are the mean percentage of hCD20^+^ cells within the CD45^+^ population ± SD of 5 spleens/treatment group. (**J**) Representative Spleen IHC for hCD20^+^ cells (brown) indicating αCD20-EndoP125A reduction of infiltrating 38c13-hCD20 lymphoma cells. *p*-value < 0.05 was considered statistically significant or marked ns for not significant. Indicated *p*-values are * < 0.05, ** < 0.01, *** < 0.001, and **** < 0.0001.

To assess hematogenous spread, circulating tumor cells (CTCs) were measured in peripheral blood on d20. CTCs were shown to be markedly decreased with αCD20-EndoP125A compared to all other treatments (Figure 6A,B). The ability of αCD20-EndoP125A to significantly reduce primary tumor growth and CTCs, as well as reduce tumor angiogenesis and lymphangiogenesis more effectively than Fc-EndoP125A, indicated the importance of the lymphoma-targeting domain of αCD20-EndoP125A.

### 3.9. αCD20-EndoP125A Reduces Lymphoma Dissemination to the TDLNs, Spleen, Lung, and Brain

Excised TDLNs from both PBS- and Rituximab-treated mice were significantly enlarged. Both Fc-EndoP125A and αCD20-EndoP125A treatment markedly reduced TDLN volume compared to Rituximab and PBS (Figure 6C,D). The composition of TDLNs, as determined by flow cytometry, indicated substantial infiltration of hCD20^+^ lymphoma cells in PBS-, Rituximab-, and Fc-EndoP125A-treated mice (Figure 6E). In contrast, αCD20-EndoP125A-treated mice demonstrated significantly reduced infiltration of hCD20^+^ lymphoma cells, and significantly increased CD4^+^ and CD8^+^ T cell infiltration within the TDLNs (Figure 6E,F). The IHC of TDLNs confirmed the αCD20-EndoP125A reduction of hCD20^+^ lymphoma cell infiltration relative to PBS-, Rituximab-, and Fc-EndoP125A-treated mice (Figure 6G).

**Figure 7:**
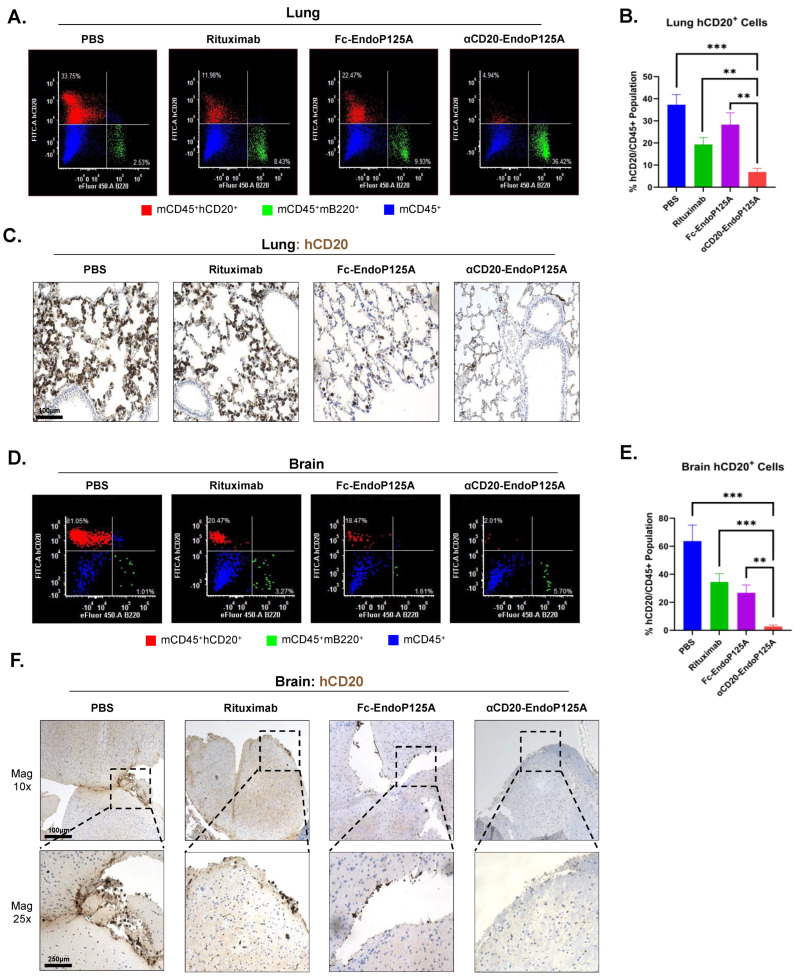
αCD20-EndoP125A significantly reduced extra-nodal lymphoma dissemination to the lung and brain: (**A**) Representative flow cytometry of the lung depicting hCD20^+^ cells (red) and B220^+^ cells (green) within the CD45^+^ cell population (blue). (**B**) Flow cytometry quantification of the percentage of infiltrating 38c13-hCD20 lymphoma cells within the lung. Results are the mean percentage of hCD20^+^ cells within the CD45^+^ population ± SD of 5 lungs/treatment group. (**C**) Representative IHC for hCD20^+^ cells (brown) indicating infiltrating 38c13-hCD20 lymphoma cells within the lung. (**D**) Representative flow cytometry of brain depicting hCD20^+^ cells (red) and B220^+^ cells (green) within the CD45^+^ cell population (blue). (**E**) Flow cytometry quantification of the percentage of infiltrating 38c13-hCD20 lymphoma cells within the brain. Results are the mean percentage of hCD20^+^ cells within the CD45^+^ population ± SD of 5 brains/treatment group. (**F**) Representative IHC for hCD20^+^ cells (brown) indicating infiltrating 38c13-hCD20 lymphoma cells within the brain. *p*-value < 0.05 was considered statistically significant or marked ns for not significant. Indicated *p*-values are * < 0.05, ** < 0.01, *** < 0.001, and **** < 0.0001.

We also investigated hCD20^+^ lymphoma cell dissemination to the spleen, lung, and brain by flow cytometry and IHC. Mice treated with αCD20-EndoP125A had significantly reduced dissemination of lymphoma cells to all three tissues (Figure 6H–J and Figure 7A–F). In patients with aggressive forms of lymphoma, such as Burkitt’s lymphoma or aggressive MCL, dissemination to the CNS has a predilection for the leptomeninges [7,54,55]. Similarly, 38c13-hCD20 CNS spread localized to the meninges. Meningeal infiltration was inhibited by αCD20-EndoP125A treatment relative to PBS-, Rituximab-, and Fc-EndoP125A-treated mice, as determined by both IHC and flow cytometry (Figure 7D–F). Of note, Jeko-1-Luc-GFP distributed similarly within the meninges of PBS-treated mice, while dissemination to the CNS was markedly inhibited by αCD20-EndoP125A treatment, as shown by flow cytometry (Supplementary Figure S9J,K).

### 3.10. αCD20-EndoP125A Inhibited Lymphoma Vessel Co-Option in the Lung

38c13-hCD20 invasion within the lung primarily populated as single-cell deposits distributed throughout the alveoli consistent with lymphoma vessel co-option (Figure 7C) and resembled growth patterns reported for solid tumors [11]. Of note, Jeko-1-Luc-GFP also distributed similarly within the lung of PBS-treated mice, while dissemination to the lung was inhibited by αCD20-EndoP125A treatment as shown by flow cytometry (Supplementary Figure S9F,G). Interstitial and lepidic deposits of lymphoma were frequently observed in PBS-, Rituximab-, and Fc-EndoP125A- but not in αCD20-EndoP125A-treated mice. We also found several instances where tumor cells had grown in an alveolar or perivascular cuffing pattern in PBS- and Rituximab-treated mice (Supplementary Figure S12C).

We, therefore, show that lymphoma cells may undergo vessel co-option in support of tumor growth within the lung; however, αCD20-EndoP125A treatment inhibits lymphoma vessel co-option and localized expansion within the lung. In summary, αCD20-EndoP125A demonstrated the ability to reduce primary tumor growth, angiogenesis, and lymphangiogenesis, as well as lymphoma vessel co-option and dissemination to the TDLNs, spleen, lung, and CNS.

## 4. Discussion

The development of new vessels, as well as the co-option of existing vasculature by lymphoma cells, supports tumor growth and progression. The spread of lymphoma or other hematologic malignancies to vital organs is frequently seen and is a leading cause of lymphoma morbidity and mortality. In addition to traditional angiogenesis, we found that lymphoma cells will migrate to and anneal with existing vascular structures, consistent with vessel co-option.

In this study, we developed a surrogate model for the study of lymphoma–vessel interactions and vessel co-option in vitro and in vivo. We also constructed a novel antibody fusion protein, αCD20-EndoP125A, for the purpose of delivering anti-angiogenic signals to lymphoma deposits. Our in vitro model predicted the activity of αCD20-EndoP125A in vivo, including inhibition of angiogenesis, lymphangiogenesis, vessel co-option, and lymphoma dissemination. The ability of our in vitro model systems to study tumor–vessel interactions and predict the potential in vivo activity of our fusion protein could be used to test other anti-angiogenic or anti-motility agents alone or in combination. The unique configuration of αCD20-EndoP125A targets CD20^+^ lymphoma cells and delivers mutant EndoP125A in a dimeric form. αCD20-EndoP125A binds both endothelial cells and CD20^+^ lymphoma cells impairing cellular migration, which are needed for endothelial vessel formation and for lymphoma vessel co-option. While we targeted CD20 in these experiments, other lymphoid targets such as CD19, CD22, CD38, or CD79b could also be used to deliver anti-angiogenic payloads.

**Figure 8:**
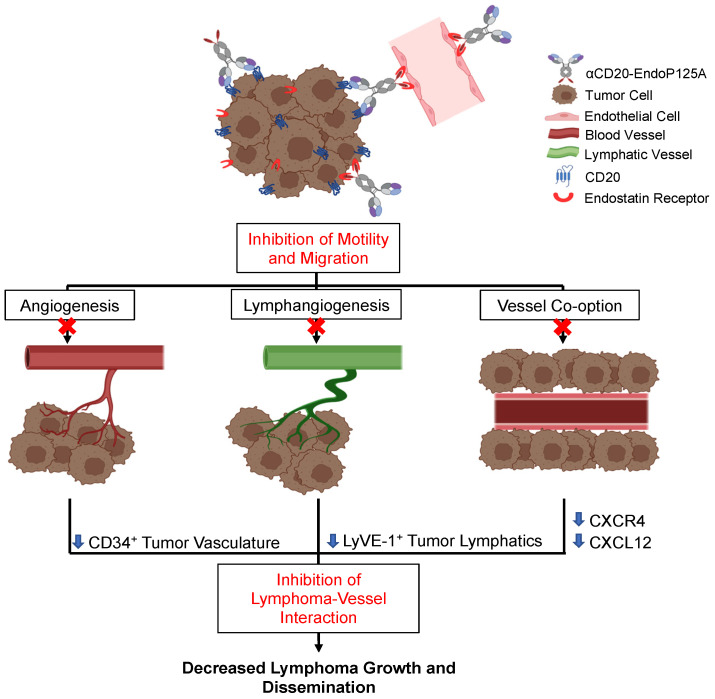
Schematic of αCD20-EndoP125A inhibition of vasculogenesis, lymphoma vessel co-option, and dissemination: αCD20-EndoP125A binds to lymphoma cells via the αCD20 and EndoP125A domains and binds to endothelial cells via the EndoP125A domain. αCD20-EndoP125A binding inhibits endothelial cell migration needed for angiogenesis and lymphangiogenesis and lymphoma cell motility and migration, preventing lymphoma vessel co-option. αCD20-EndoP125A suppression of CXCL12 and CXCR4 expression by both endothelial and lymphoma cells contributes to the inhibition of vessel formation and prevention of lymphoma interaction with vessels. In vivo treatment with αCD20-EndoP125A markedly reduced CD34^+^ tumor vasculature and LyVE-1^+^ tumor lymphatics, thereby decreasing lymphoma growth and dissemination.

Lymphoma dissemination may involve intravasation for entry into the circulation and/or lymphatics, and extravasation for invasion into tissue. αCD20-EndoP125A reduced CTCs, homing to regional lymph nodes and the spleen, and extra-nodal hematogenous dissemination to the lungs and brain, suggesting that both intravasation and extravasation were inhibited. Rituximab does not inhibit 38c13-hCD20 primary tumor growth or directly induce apoptosis, however, rituximab can mediate both ADCC and CDC against 38c13-hCD20 cells in an immunocompetent environment [53]. In our in vivo model, 38c13-hCD20 cells were implanted into C3H immunocompetent mice and the cytotoxic effects of Rituximab may contribute to the observed reduction in CTCs. αCD20-EndoP125A demonstrated overall enhanced anti-tumor activity relative to Rituximab and retained the ability to induce antibody-mediated cytotoxicity. The enhanced anti-tumor activity of αCD20-EndoP125A may be due to engagement of both lymphoma and endothelial cells, reducing vascular support through effects on angiogenesis, lymphangiogenesis, and lymphoma vessel co-option, leading to more effective inhibition of lymphoma growth and dissemination (Figure 8).

The CXCL12–CXCR4 axis promotes both endothelial and tumor cell migration, as well as tumor vascularization [48,56,57]. Previous studies have shown that CXCR4 expression is essential for endothelial cellular polarization and that receptor interaction with its CXCL12 ligand induces intracellular actin polymerization, both of which are necessary for endothelial cell motility, branching, and vessel formation [58]. We demonstrate induction of both CXCL12 and CXCR4 expression by HUVEC cells forming CLS and by MCL cells undergoing vessel co-option. Treatment with chemokine-neutralizing antibodies specific to CXCL12 and CXCR4 markedly inhibited CLS formation, which was further reduced by non-specifically targeting all chemokine receptors with pertussis toxin. After treatment with chemokine inhibitors, the HUVEC cells appeared isolated or assembled into cell aggregates of short thin cords that did not connect to form complete tubule networks. In contrast, treatment with αCD20-EndoP125A completely inhibited CLS formation and the HUVEC cells formed monolayers. αCD20-EndoP125A also inhibited MCL vessel co-option more effectively than selective chemokine inhibition or pertussis toxin. αCD20-EndoP125A reduced MCL CXCR4 expression more significantly than Rituximab and Fc-EndoP125A treatment. Additionally, the reduction in MCL vessel co-option with Rituximab or Fc-EndoP125A treatment was similar to treatment with αCXCR4 or αCXCL12 antibodies. A reduction in the overall motility of MCL cells and decreased CXCR4 expression could lower responsiveness to CXCL12 and contribute to the observed inhibition of MCL vessel co-option.

In addition to CXCL12 and CXCR4, we found several angiogenic growth factors, including EGF, PDGF, FGF-2, and VEGF-A, which were upregulated in untreated HUVEC and HUVEC:MCL cocultures on Matrigel (Supplementary Figure S5E). It was reported that increases in expression of bFGF and VEGF induced CXCR4 expression on endothelial cells. The interaction of CXCL12 with CXCR4^+^ endothelial cells may in turn lead to further expression of bFGF, VEGF, and CXCL12, resulting in a pro-angiogenic feedforward loop [58,59,60]. In addition to promoting angiogenesis, this loop may also facilitate lymphoma migration to, and interaction with, supporting vasculature (vessel co-option). αCD20-EndoP125A significantly reduced CXCL12 and CXCR4 expression, as well as several pro-angiogenic growth factors, resulting in disruption of the CXCL12/CXCR4 signaling loop and inhibition of vessel formation and co-option (Supplementary Figure S8). Specifically targeting chemokine–chemokine receptor interactions contributes to the inhibition of vessel formation and vessel co-option but does not fully reproduce the extensive morphological changes seen with αCD20-EndoP125A. Expanded investigation into pathways implicated in endostatin activity and vessel co-option such as WNT/beta catenin and integrin signaling, may shed further light onto the effects of lymphoma motility, migration, and vessel co-option that we observed.

αCD20-EndoP125A is a novel therapy which inhibits key physiologic processes linked to lymphoma progression, including angiogenesis, lymphangiogenesis, lymphoma vessel co-option, cytokine/chemokine signaling and lymphoma cell migration and invasion. Prevention of extra-nodal spread and dissemination to the CNS may be especially important in reducing lymphoma morbidity. Vessel co-option has been implicated in tumor initiation, metastatic spread, and resistance to anti-angiogenics. The ability of αCD20-EndoP125A to simultaneously inhibit both lymphoma vessel formation and vessel co-option that may provide a potent means of inhibiting lymphoma progression and therapeutic resistance. While this study utilized an anti-CD20 antibody, this strategy has worked for several different malignancies and could be adapted to a variety of hematologic targets. αCD20-EndoP125A has a unique ability to target lymphoma motility and deliver anti-vasculogenic signals which inhibit angiogenesis, lymphangiogenesis, and lymphoma vessel co-option. The combined effects offer a novel ‘migrastatic’ therapeutic strategy for the inhibition of lymphoma growth and extra-nodal dissemination, with the potential to substantially impact morbidity and mortality.

## Data Availability

All data generated and analyzed during this study are included within this published article.

## Supplementary Materials

The following supporting information can be downloaded at: https://www.mdpi.com/article/10.3390/cells13221835/s1, Figure S1: MCL cell lines randomly disperse when cultured alone but migrate to and align with HUVEC capillary-like structures (CLS) in coculture; Figure S2: αCD20-EndoP125A does not induce lymphoma apoptosis alone but mediates ADCC in the presence of NK effector cells; Figure S3: αCD20-EndoP125A inhibits CLS formation more effectively than bevacizumab; Figure S4: αCD20-EndoP125A reduces MCL and endothelial cell migration and invasion; Figure S5: Expression of chemokines, chemokine receptors and pro-angiogenic growth factors and reduction of expression by αCD20-EndoP125A; Figure S6: CXCR4 Gating; Figure S7: Chemokine inhibitors αCXCL12 αCXCR4, or pertussis toxin impair CLS formation; Figure S8: Angiogenic and Vessel Co-option Signaling Loop; Figure S9: αCD20-EndoP125A reduces Jeko-1 GFP-Luciferase tumor growth and dissemination; Figure S10: 38c13-hCD20 aligns and associates with murine C166 CLS, antibody binding to 38c13-hCD20 cells, and αCD20-EndoP125A inhibition of murine CLS formation and alignment of 38c13-hCD20; Figure S11: hCD20 Gating; Figure S12: Reduced vascularity of primary tumors by αCD20-EndoP125A and types of vessel co-option within the lung; Table S1: Flow Cytometry Antibody List for Chemokine Receptor Expression; Table S2: Chemokines and Angiogenic Analytes Panel; Table S3: Flow Cytometry Antibody List for Tissues Analysis of 38c13-hCD20 in vivo Experiment.
