# Supplementary material for: Modeling Lymphoma Angiogenesis, Lymphangiogenesis, and Vessel Co-Option, and the Effects of Inhibition of Lymphoma–Vessel Interactions with an αCD20-EndoP125A Antibody Fusion Protein
Source: Cells. 2024 Nov 6;13(22):1835. doi: 10.3390/cells13221835 (PMC11593053; doi:10.3390/cells13221835)
Supplement: Supplementary file 1 [file cells-13-01835-s001.zip › cells-3251397-supplementary.pdf]

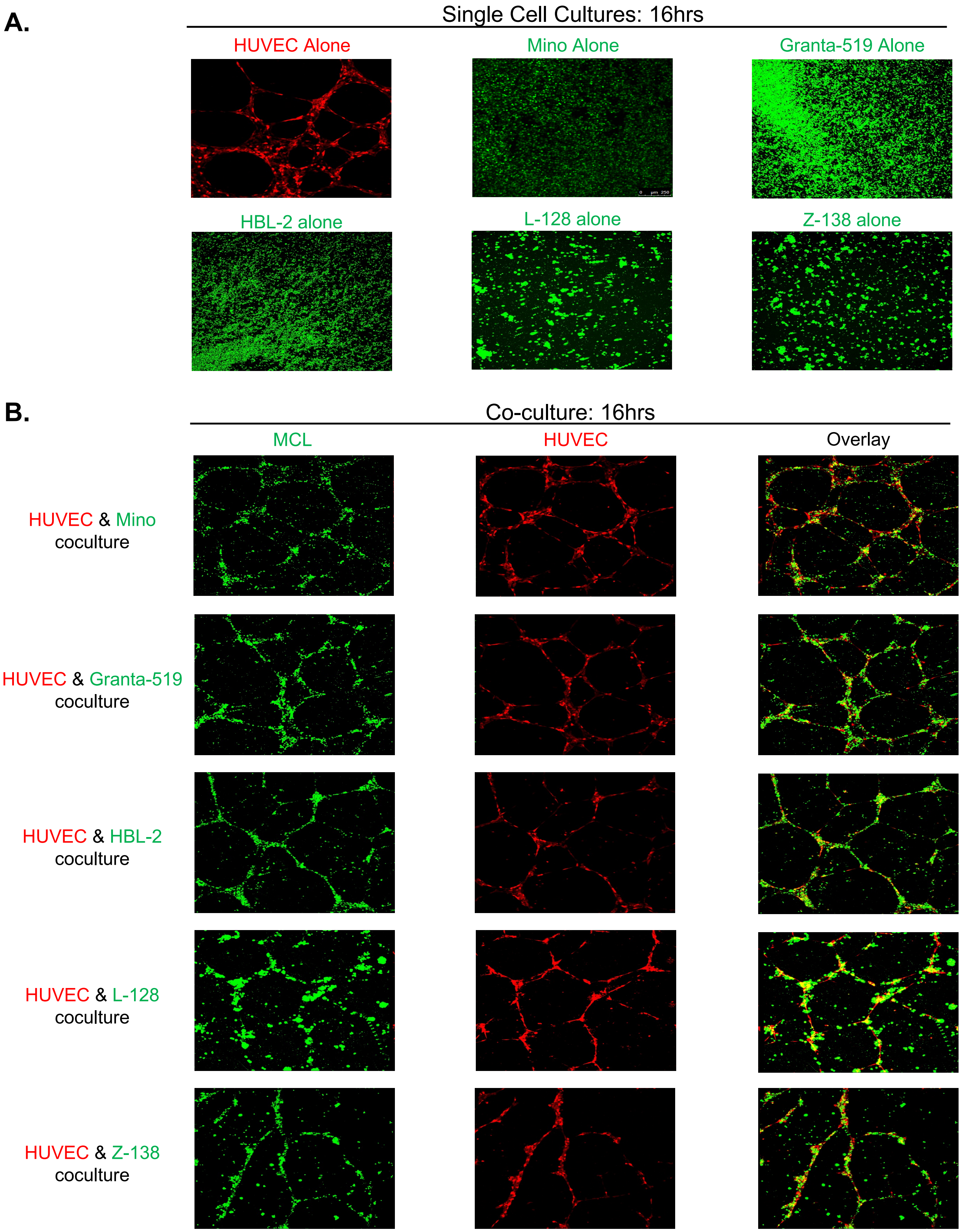

Supplementary Figure S1: MCL cell lines randomly disperse when cultured alone but migrate to and align with HUVEC capillary-like structures (CLS) in coculture MCL cell lines Mino, Granta-519, HBL-2, L-128, and Z-138 were generously provided by Dr. Izidore Lossos. To study MCL interaction with vessel-like structures, HUVEC were labelled with calcein red (Invitrogen, Cat. no C34851) and MCL with calcein green (Invitrogen, Cat. no C34852). Cells were either cultured alone or cultured in a 1:1 ratio on Matrigel. (A,B) HUVEC (red) and indicated MCL cell lines (green) were either cultured alone or co-cultured on Matrigel (A) Cultured alone HUVEC cells form CLS and MCL cells randomly disperse (B) MCL cells co-cultured with HUVEC associated and aligned with CLS

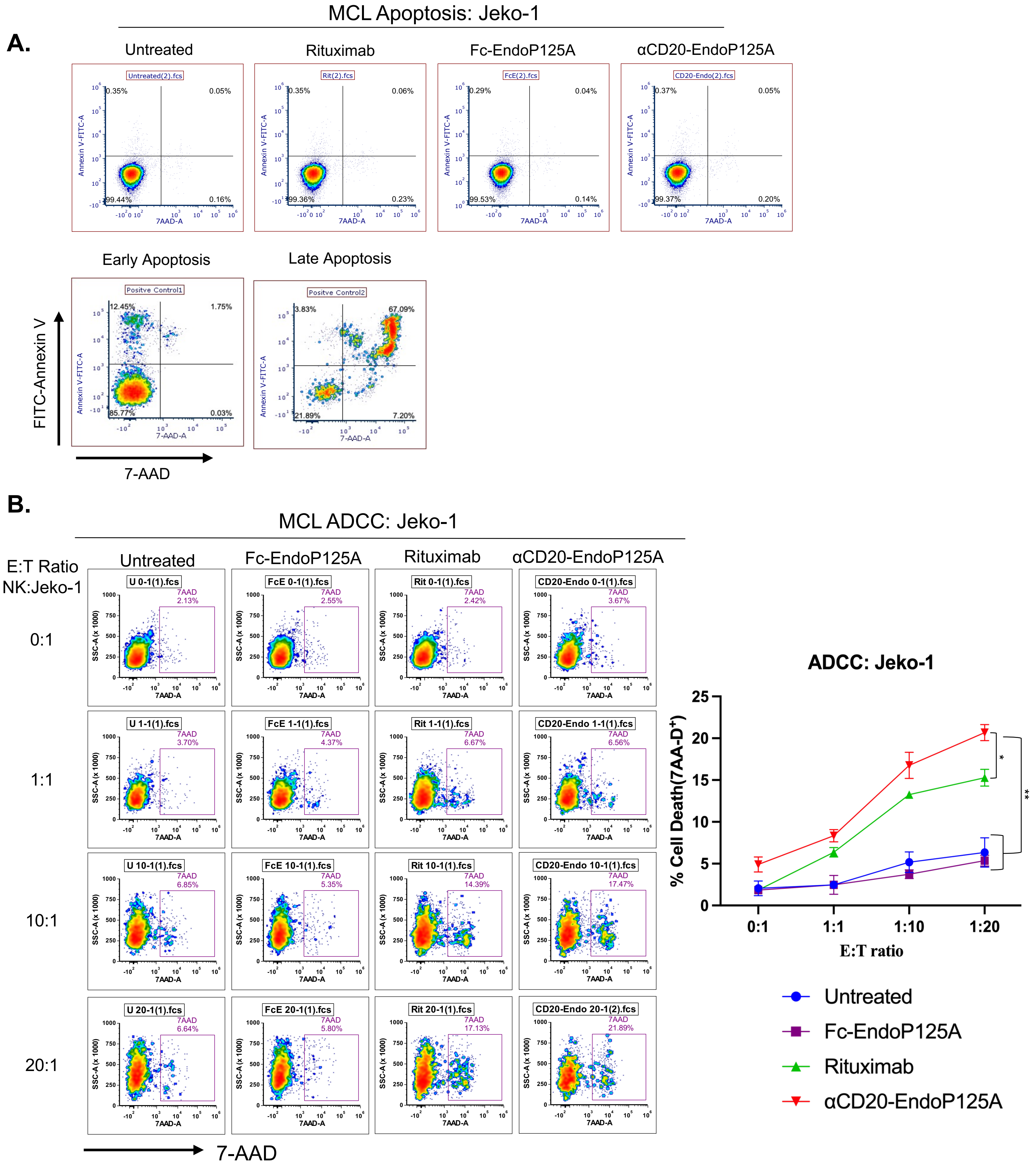

Supplementary Figure S2: αCD20-EndoP125A does not induce lymphoma apoptosis alone but mediates ADCC in the presence of NK effector cells Jeko-1 target cells were left untreated or treated as indicated with equimolar concentrations of Fc-EndoP125A(3.5μg/mL), Rituximab(6.8μg/mL), or αCD20-EndoP125A(10μg/mL) at 37ºC for 6hrs. (A) Flow cytometry for Annexin-V-FITC and 7AAD of Jeko-1 cells treated with rituximab, Fc-EndoP125A or αCD20-EndoP125A depicts little/no increase in apoptosis among the treatments. Apoptosis for positive controls was induced by heating cells for 15 seconds at 100^0C for early apoptosis and 45 seconds for late apoptosis. (B) ADCC experiments were performed with NK cells purified from human PBMC using NK cell isolation kit (Stem Cell, Cat#17955) and used as effector cells. Assays were performed in triplicate. Effector to target cell ratios are as indicated. After 6hrs incubation, cells were recovered, stained for CD19 to identify Jeko-1 cells and Annexin-V and 7AAD to identify dead cells. Flow cytometry was performed, and cell death was quantified by percent $7AAD^+$. Representative flow cytometry plots are presented and results are mean cell death ± SD.

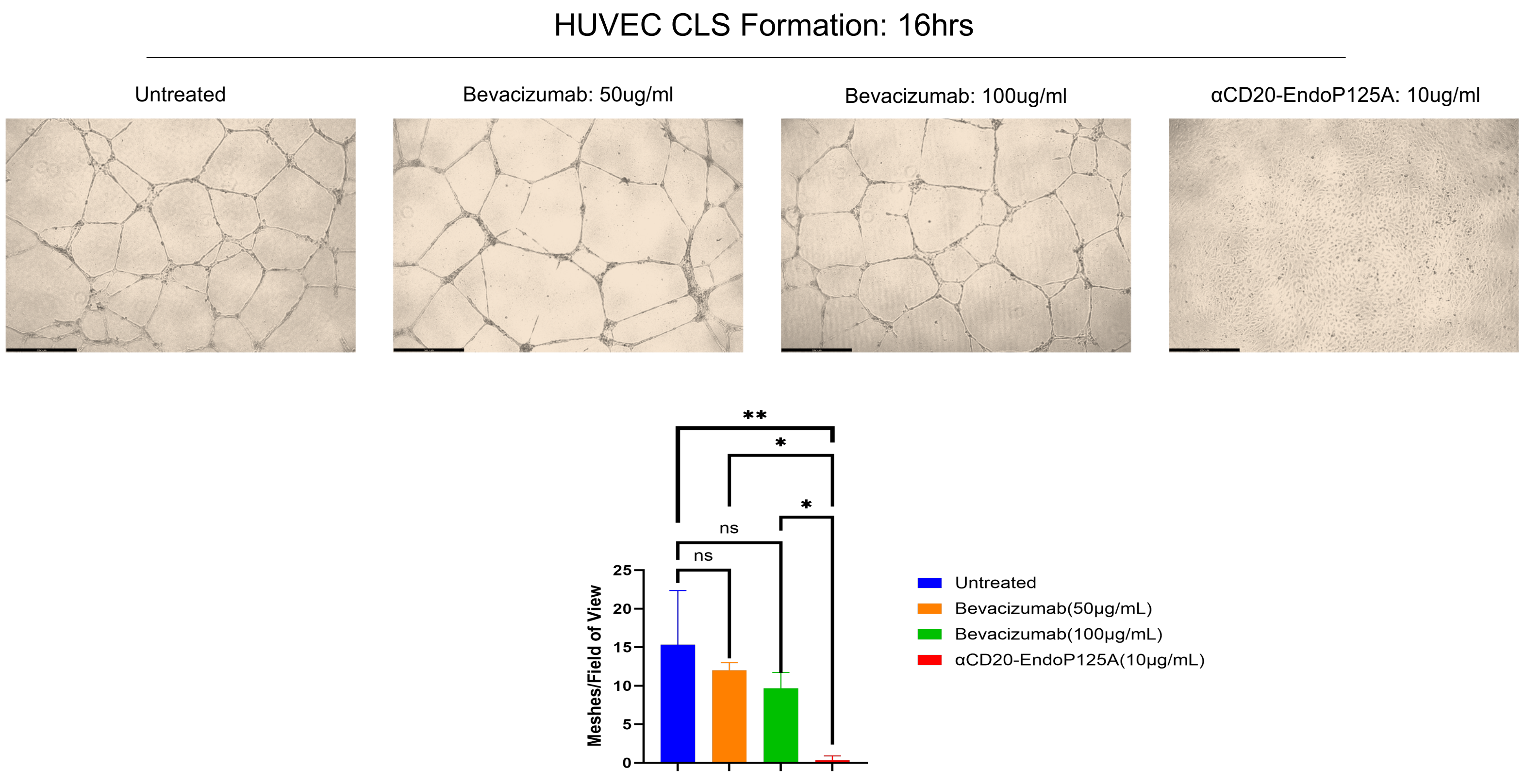

Supplementary Figure S3: αCD20-EndoP125A inhibits CLS formation more effectively than bevacizumab: HUVEC cells were cultured on Matrigel for 16hrs either untreated or treated with bevacizumab or αCD20-EndoP125A at indicated treatment concentrations. Treatments were performed in triplicate and tubule meshes were used to quantify amount of CLS formation. Results are mean number of meshes per 10x field of view ± SD. *T-test statistics were calculated and significant differences compared with control are indicated with p-values * < 0.05, ** < 0.01, *** < 0.001, **** < 0.0001

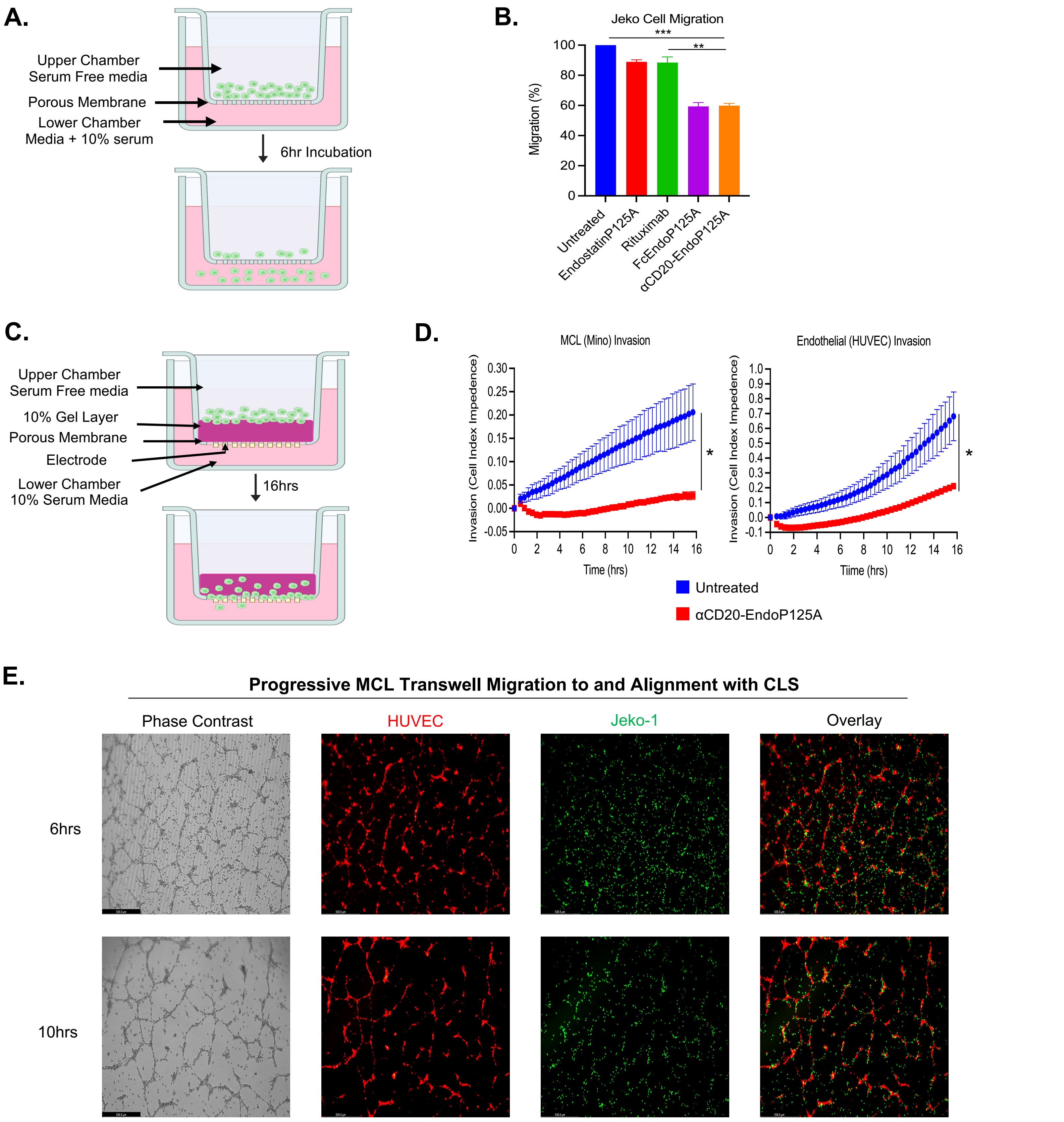

Supplementary Figure S4: **αCD20-EndoP125A reduces MCL and endothelial cell migration and invasion** (A) Schematic rendering of transwell migration assay. 10% FBS/EGM-2 media was placed in bottom chamber of a 24-well plate and a 5um porous membrane (Sterlitech SKU 9325012) separated top and bottom. MCL cells were labelled with calcein green and resuspended in serum free RPMI media. 2x10^5 labelled MCL cells in serum free media were seeded to the top chamber. Cultures in triplicate were either left untreated or treated with equimolar concentrations of EndoP125A(2µg/mL), Rituximab(6.8µg/mL), Fc-EndoP125A(3.5µg/mL), or αCD20-EndoP125A(10µg/mL). Plates incubated for 6 hours at 37°C and top chamber containing non-migrating cells was removed (B) Quantification of transwell migration was determined by fluorescent readout of bottom chamber. The untreated fluorescent readout was used as 100% migration and percent migration was calculated as mean fluorescence of treated samples divided mean fluorescence of untreated samples (C) Schematic rendering of transwell invasion assay. Transwell inserts were coated with 10% Matrigel. HUVEC or MCL cells were seeded in top chamber in serum free media and bottom chamber contained 10% FBS/EGM-2 to act as a chemoattractant. Cells were incubated for 16 hours and either left untreated or treated with αCD20-EndoP125A. (D) Impedance from the plate sensor was used as a measure invasion over time using the Xcelligence system (E) Progressive migration of MCL cells labeled with calcein green from top chamber of transwell to bottom chamber and alignment with HUVEC CLS labeled with calcein red and cultured in Matrigel. Phase contrast and fluorescent images at 6hr and 10hr time points are shown . *T-test statistics were calculated and significant differences compared with control are indicated with p-values * < 0.05, ** < 0.01, *** < 0.001, **** < 0.0001

Supplementary Figure S5 Methods

Chemokines, Chemokine Receptors and Angiogenic Protein Expression Assays

We investigated chemokine-chemokine receptor interactions between lymphoma and endothelial cells to identify chemokines that could facilitate (1) endothelial vessel formation, (2) attraction of lymphoma cells to vessels, and (3) chemokines that αCD20-EndoP125A could be affecting to inhibit such interactions

5 A,B: Chemokine Dot Blot Array

Jeko-1 or HUVEC cells were cultured on Matrigel either alone or in coculture. Cell cultures were either left untreated or treated with αCD20-EndoP125A(10μg/mL) and incubated for 6hrs at 37°C. Cell culture supernatant was collected, and dot blot array (Abcam, cat#169812) was produced per manufacturer's protocol. Relative chemokine expression was calculated by normalizing dot blot area to negative and positive reference control spots per protocol manual.

5C: Chemokine Receptor Expression Assay

Jeko-1 cells were blocked with Human TruStain FcX (Biolegend cat#422302) for 10min at room temperature then stained with antibodies (Antibody List Supp. Table 1) for 30 minutes at 4°C in the dark. Samples were washed with PBS, resuspended in 500uL of staining buffer (1x PBS: 0.5%BSA, 2mM EDTA, 0.09%SA) and flow cytometry was performed on a Cytek Aurora Spectral Analyzer

Table S1: Flow Cytometry Antibody List for Chemokine Receptor Expression

Target	Fluorescence	Vendor	Catalog #
CCR1	Per CP-Cy5.5	BioLegend	362911
CCR2	BV785	BioLegend	357233
CCR3(CD193)	BUV737	BD Biosciences	612853
CCR4(CD194)	BUV395	BD Biosciences	744142
CCR5	PE CY7	BioLegend	359107
CCR8	PE	BioLegend	365803
CCR6	BV605	BioLegend	353419
CCR7	BV711	BioLegend	353227
CXCR1(CD181)	BUV496	BD Biosciences	750747
CXCR2(CD182)	BUV615	BD Biosciences	751242
CXCR3	BV510	BioLegend	353725
CXCR4	BUV563	BD Biosciences	741400
CXCR5 (CD185)	R718	BD Biosciences	752012
CXCR6	APC	BioLegend	356005
CXCR7	BV421	BD Biosciences	566234
CX3CR1	BUV805	BD Biosciences	749353
CD45	APC-Fire750	Biolegend	304062
CD31	FITC	Invitrogen	BMS137FI
Live dead blue		Thermofisher	L34957

5 E: ProcartaPlex Multi-Analyte Protein Assay

98 different chemokines and angiogenic proteins were investigated (Supp. Table 2). Jeko-1 or HUVEC cells were cultured in media or on Matrigel either alone or in co-culture. Cell cultures were left untreated or treated with equimolar concentrations of Fc-EndoP125A(3.5μg/mL), Rituximab(6.8μg/mL), or αCD20-EndoP125A(10μg/mL) and incubated for 6hrs at 37°C. Cell culture supernatant was collected and ProcartaPlex assays were followed according to protocol (Thermofisher, cat#EPX800-10080-901, cat#EPX180-15806-901, cat#PPX-06). Each individual analyte in ProcartaPlex system was captured with specific detection antibody conjugated to a different fluorescent bead. This allowed for simultaneous quantification of each individual analyte using mean fluorescence readout. Flow cytometry was performed on a FlexMap 3D. Analysis and conversion of MFI to protein concentration(pg/mL) was performed on Thermofisher's ProcartaPlex app

Table S2: Chemokines and Angiogenic Analytes Panel

1	6Ckine (CCL21)	50	IL-17A (CTLA-8)
2	APRIL	51	IL-18
3	Angiopoietin-1	52	IL-20
4	Angiostatin	53	IL-21
5	BAFF	54	IL-22
6	BLC (CXCL13)	55	IL-23
7	BMP-9	56	IL-27
8	CD30	57	IL-31
9	CD31 (PECAM-1)	58	IL-34
10	CD40L	59	IL-37
11	EGF	60	IP-10 (CXCL10)
12	EMMPRIN	61	Leptin
13	ENA-78 (CXCL5)	62	LIF
14	Eotaxin (CCL11)	63	LYVE-1
15	Eotaxin-2(CCL24)	64	M-CSF
16	Eotaxin-3 (CCL26)	65	MCP-1 (CCL2)
17	FGF-2	66	MCP-2(CCL8)
18	Follistatin	67	MCP-3 (CCL7)
19	Fractalkine(CX3CL1)	68	MCP-4 (CCL13)
20	G-CSF (CSF-3)	69	MDC
21	GCP-2 (CXCL6)	70	MIF
22	GMCSF	71	MIG (CXCL9)
23	GRO alpha (CXCL1)	72	MIP-1 alpha (CCL3)
24	Galectin-3	73	MIP-1 beta(CCL4)
25	Granzyme A	74	MIP-2 alpha (CXCL2)
26	Granzyme B	75	MIP-3 alpha (CCL20)
27	HBEGF	76	MIP-3 beta (CCL19)
28	HGF	77	MIP-4 (CCL18)
29	HMGB1 (HMG-1)	78	MMP-1
30	I-309 (CCL1)	79	MPIF (CCL23)
31	I-TAC (CXCL11)	80	NGF beta
32	IFN alpha	81	PDGF-BB
33	IFN gamma	82	PTX3
34	IL-1 alpha	83	RANTES (CCL5)
35	IL-1 beta	84	SCF
36	IL-2	85	SDF-1 alpha(CXCL12)
37	IL-2R	86	Syndecan
38	IL-3	87	TARC (CCL17)
39	IL-4	88	TECK (CCL25)
40	IL-5	89	TIE-2
41	IL-6	90	TNF alpha
42	IL-7	91	TNF beta
43	IL-8 (CXCL8)	92	TNF-RII
44	IL-9	93	TRAIL
45	IL-10	94	TREM-1
46	IL-12p70	95	TSLP
47	IL-13	96	TWEAK
48	IL-15	97	VEGF-A
49	IL-16	98	VEGF-D

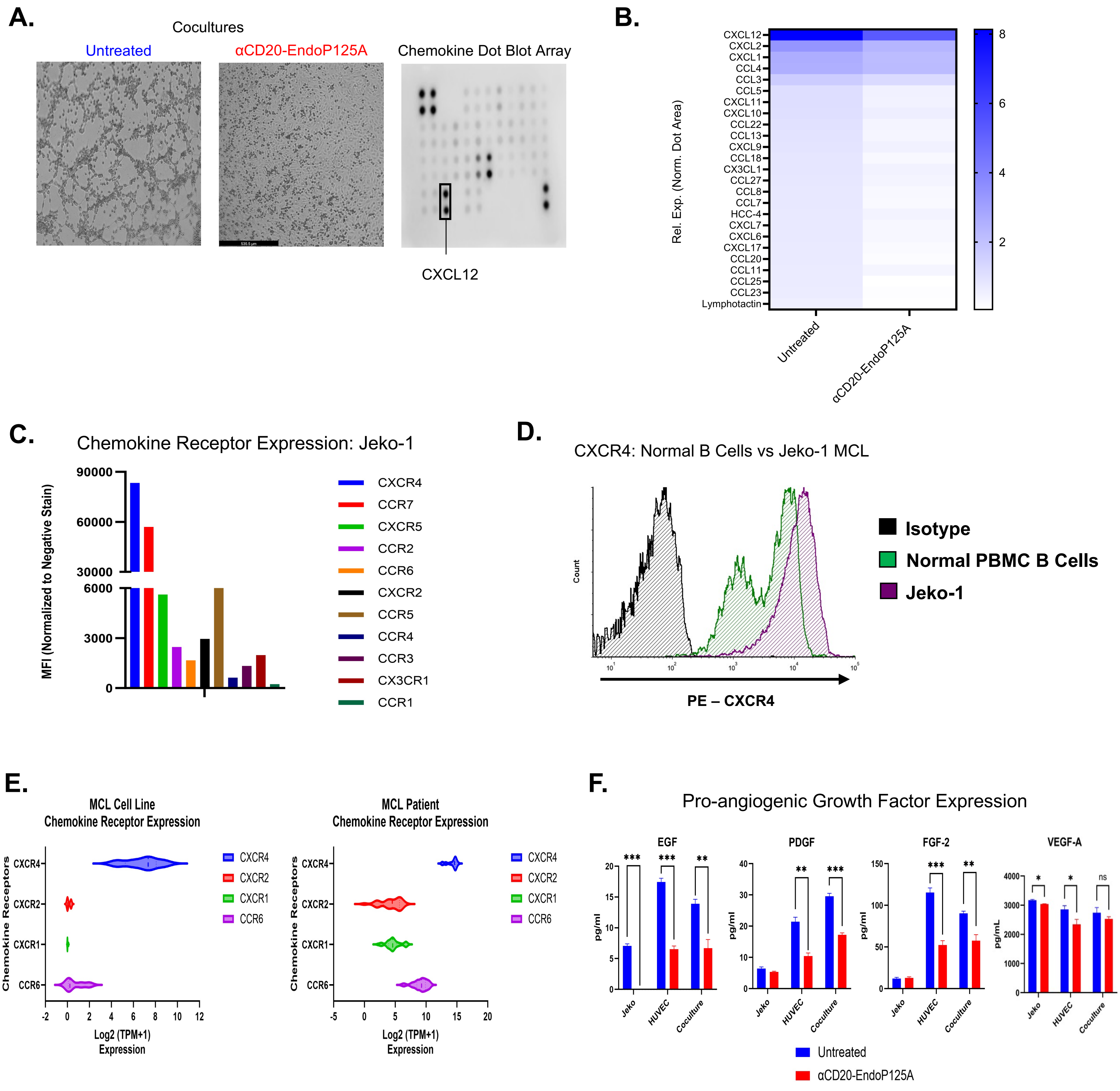

Supplementary Figure S5: Expression of chemokines, chemokine receptors and pro-angiogenic growth factors and reduction of expression by αCD20-EndoP125A (A) Jeko-1 and HUVEC cells were co-cultured for 6hrs on Matrigel and either left untreated or treated with αCD20-EndoP125A. Supernatant was collected and incubated on chemokine dot blot membrane array. Representative dot blot image displayed (B) Relative chemokine expression was performed by normalizing dot blot area to positive and negative control dots and heatmap plotted (C) Jeko-1 chemokine receptor expression determined by flow cytometry, MFI normalized to negative stain plotted (D) Jeko-1 cells express increased CXCR4 compared to normal human B cells purified from PBMC (E) mRNA expression of chemokine receptors of interest for 5 MCL cell lines and 28 MCL patients in DepMap and GENT2 databases indicating CXCR4 to be highly expressed (F) Jeko-1 and HUVEC cells were cultured alone or co-cultured for 6hrs on Matrigel and either left untreated or treated with αCD20-EndoP125A. Supernatant was collected and procarta plex multi-analyte assay was performed to determine angiogenic growth factor expression. Increased expression among untreated samples was seen for angiogenic growth factors EGF, PDGF, FGF-2, and VEGF-A in which expression was markedly reduced by αCD20-EndoP125A treatment. Assays performed in triplicate. Results are mean ± S.D. *T-test statistics were calculated and significant differences compared with control are indicated with p-values * < 0.05, ** < 0.01, *** < 0.001, **** < 0.0001

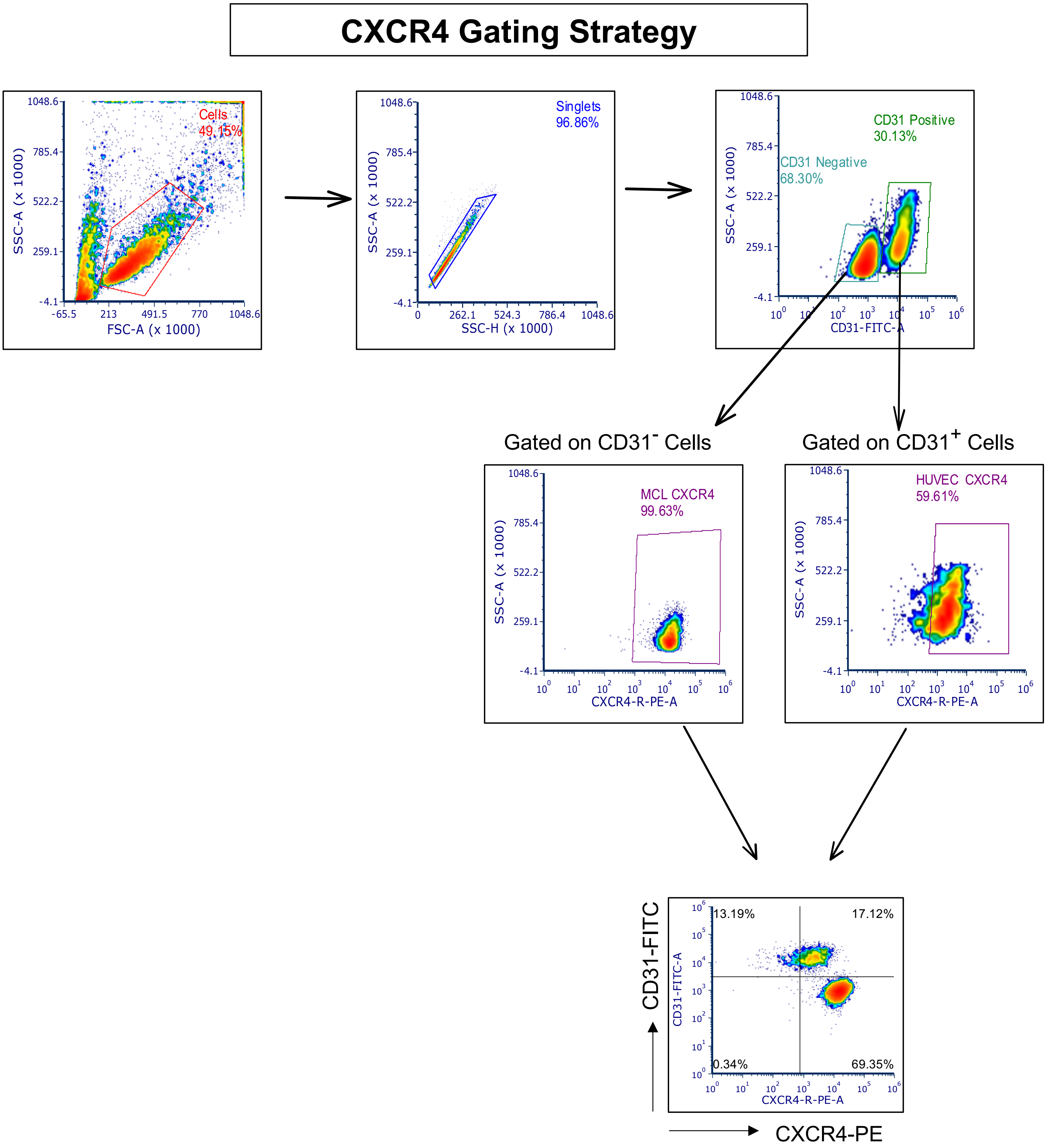

Supplementary Figure S6: CXCR4 Gating - Flow cytometry gating strategy used to distinguish MCL and HUVEC cells and determine CXCR4 expression

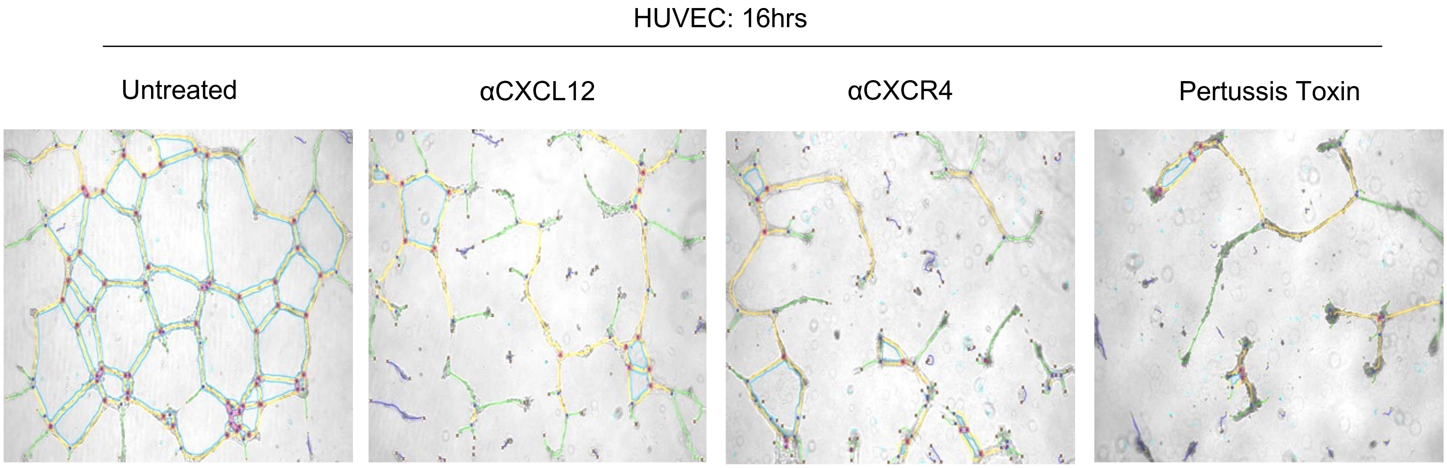

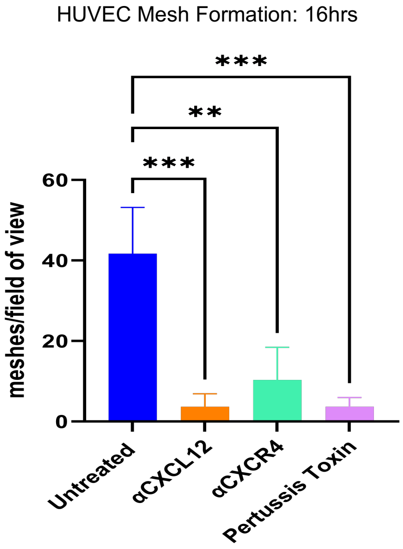

Supplementary Figure S7: Chemokine inhibitors αCXCL12 αCXCR4, or pertussis toxin impair CLS formation. HUVEC cells were cultured on Matrigel for 16hrs and either left untreated or treated with αCXCL12(10μg/mL), αCXCR4(10μg/mL), or Pertussis Toxin(200ng/mL). Red dots indicates nodes, yellow line indicates branches, and blue polygons indicate intact tubule mesh formed. Tubule meshes were used to quantify CLS formation for triplicate wells per treatment. Results are mean number of meshes ± SD

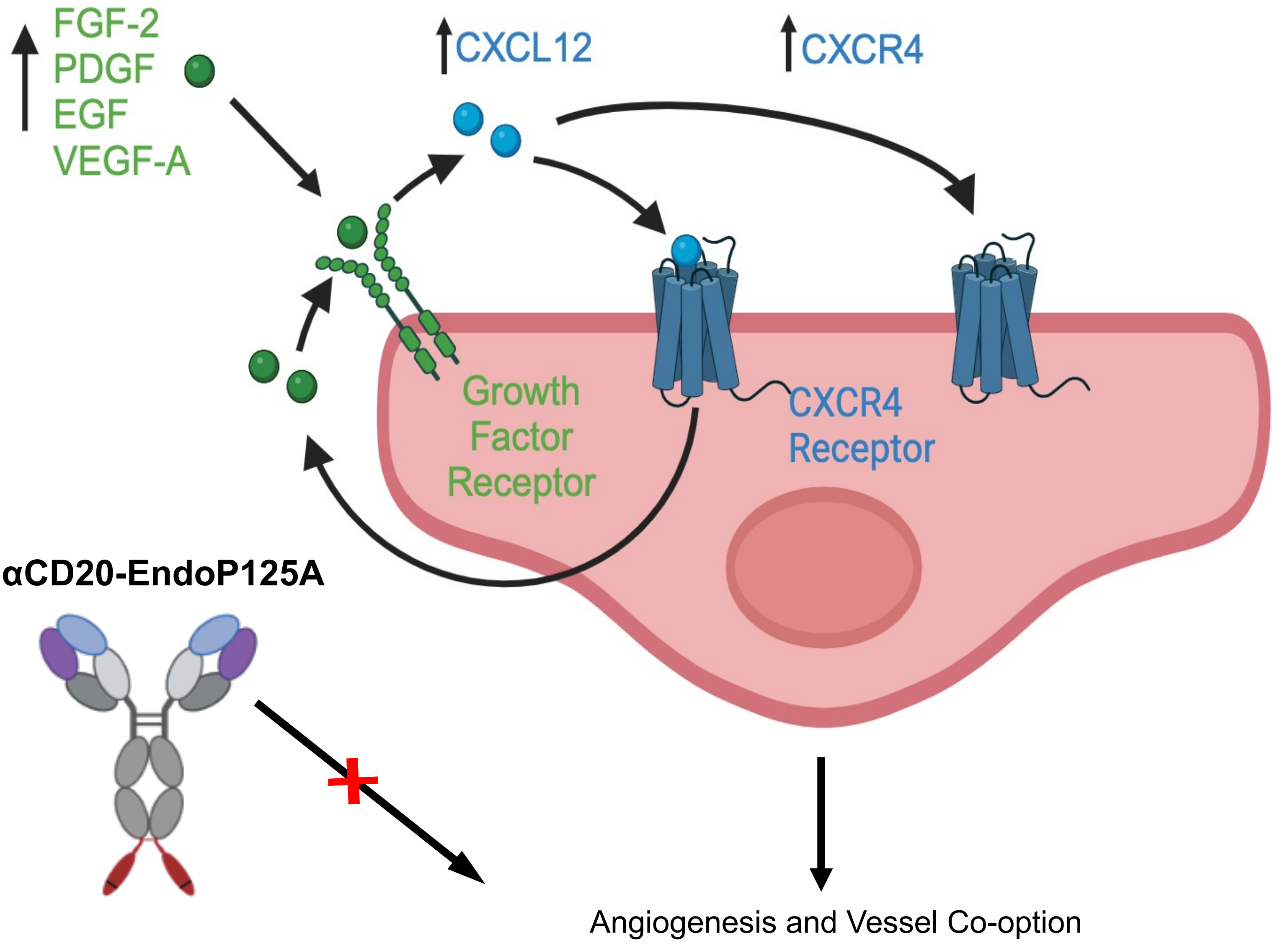

Supplementary Figure S8: Angiogenic and Vessel Co-option Signaling Loop: Growth factors, including FGF-2, PDGF, EGF-2, and VEGF-A which promote vessel formation and maturation were expressed at high levels when HUVEC cells were cultured on Matrigel undergoing CLS formation and when MCL cells were cultured on Matrigel undergoing vessel co-option. Increases in growth factor expression coincided with increased CXCL12 expression in the supernatant and increased CXCR4 expression on both MCL and HUVEC cells. Salcedo and Oppenheim[1] reported an angiogenic feed forward loop in which increases in expression of bFGF and VEGF induced CXCR4 expression on endothelial cells. The $CXCR4^{+}$ endothelial cells will then migrate toward CXCL12 and CXCL12 interaction with CXCR4 will in turn lead to further bFGF, VEGF, and CXCL12 expression causing a feed forward loop which promotes angiogenesis. Our results suggest that this loop may promote angiogenesis and may also facilitate lymphoma migration to, and interaction with supporting vasculature. However, αCD20-EndoP125A treatment suppressed CXCL12 and CXCR4 expression, reduced expression of growth factors, and inhibited angiogenesis, and lymphoma vessel co-option.

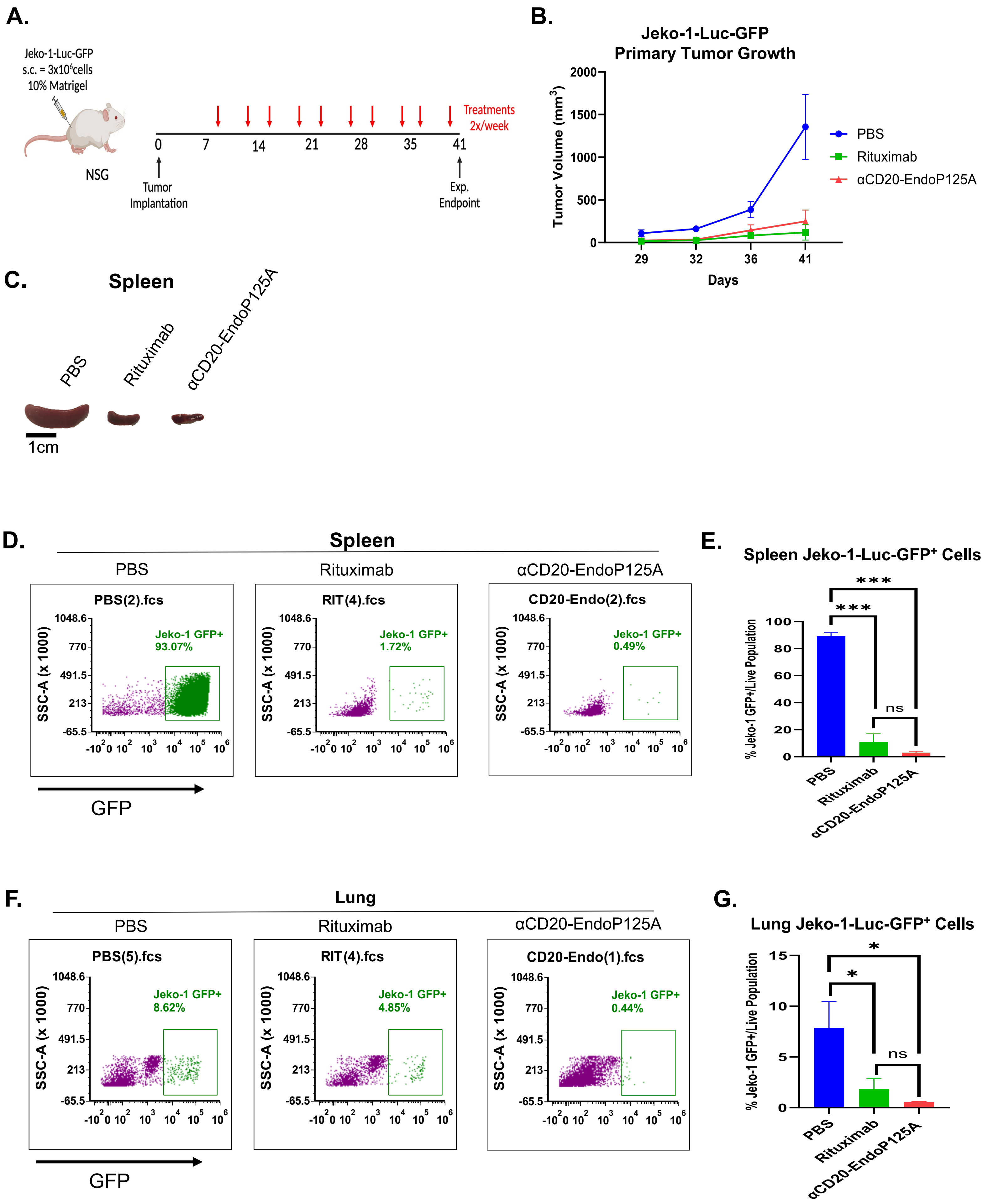

A.
Jeko-1-Luc-GFP
s.c. = 3x10⁶cells
10% Matrigel
NSG
Treatments
2x/week
0
7
14
21
28
35
41
Tumor
Implantation
Exp.
Endpoint
B.
Jeko-1-Luc-GFP
Primary Tumor Growth
Tumor Volume (mm³)
Days
PBS
Rituximab
αCD20-EndoP125A
C.
Spleen
PBS
Rituximab
αCD20-EndoP125A
1cm
D.
Spleen
PBS
Rituximab
αCD20-EndoP125A
PBS(2).fcs
RIT(4).fcs
CD20-Endo(2).fcs
Jeko-1 GFP+
93.07%
Jeko-1 GFP+
1.72%
Jeko-1 GFP+
0.49%
SSC-A (x 1000)
GFP
E.
Spleen Jeko-1-Luc-GFP⁺ Cells
% Jeko-1 GFP+/Live Population
***
***
ns
PBS
Rituximab
αCD20-EndoP125A
F.
Lung
PBS
Rituximab
αCD20-EndoP125A
PBS(5).fcs
RIT(4).fcs
CD20-Endo(1).fcs
Jeko-1 GFP+
8.62%
Jeko-1 GFP+
4.85%
Jeko-1 GFP+
0.44%
SSC-A (x 1000)
GFP
G.
Lung Jeko-1-Luc-GFP⁺ Cells
% Jeko-1 GFP+/Live Population
*
*
ns
PBS
Rituximab
αCD20-EndoP125A

H.

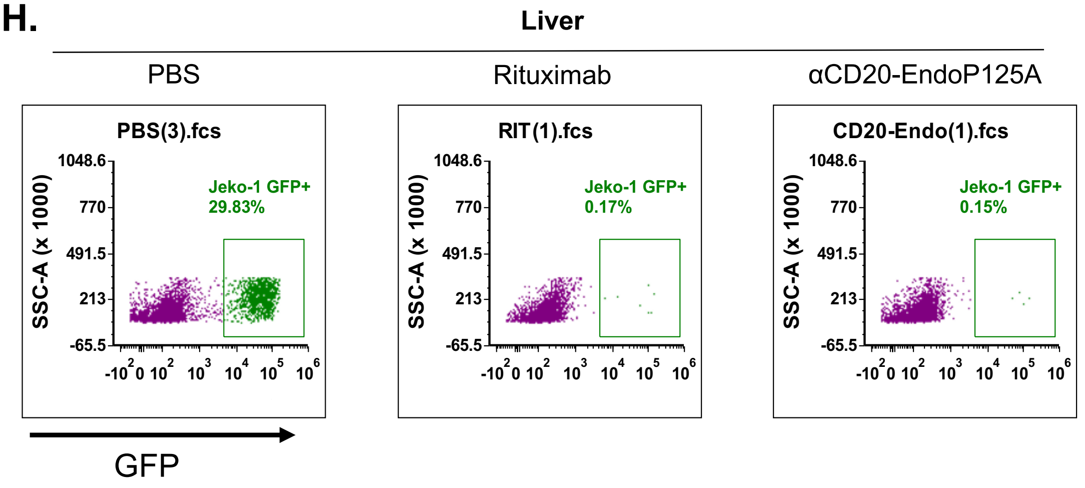

I.

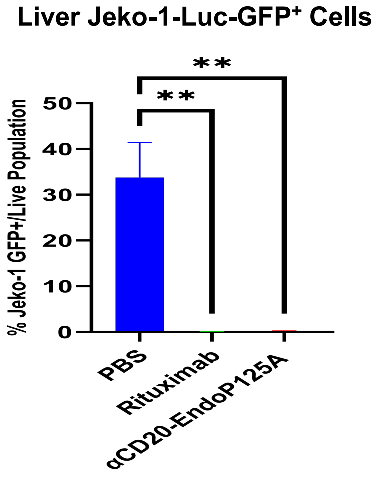

J.

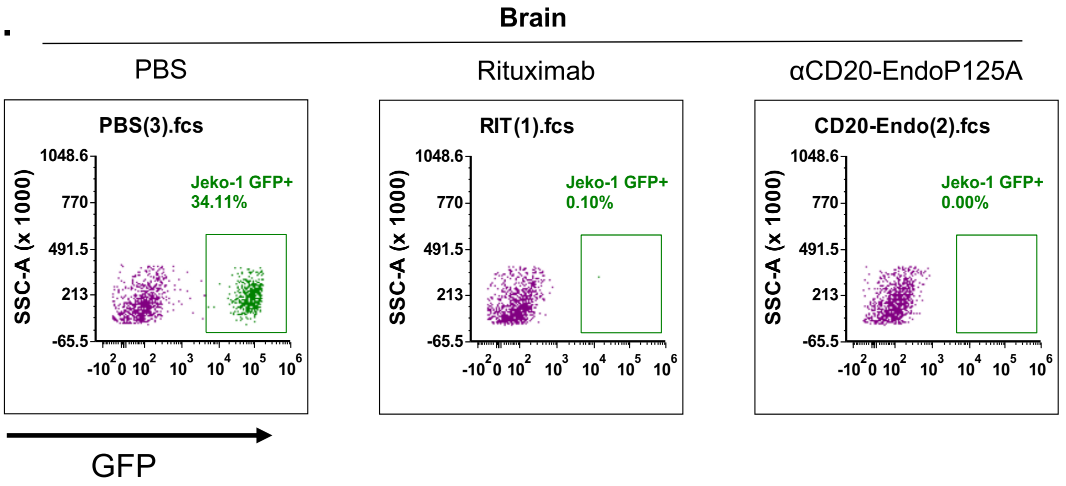

K.

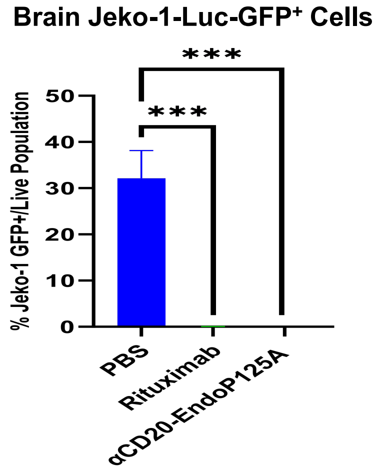

Supplementary Figure S9: αCD20-EndoP125A reduces Jeko-1 GFP-Luciferase tumor growth and dissemination. Jeko-1 cells were stably transduced to express Luciferase/GFP. Details below. (A) Schematic rendering of *in vivo* experiment. $3x10^6$ Jeko-1-Luc-GFP^+ cells suspended in 200µl PBS containing 10% Matrigel were implanted subcutaneously into right hind flank of NSG mice (female/6week, Jackson Laboratories). Starting d14 post implantation, mice were intraperitoneally treated 2x/week with 200µl PBS, or PBS containing equimolar concentrations of Rituximab (136µg/200µl) or αCD20-EndoP125A (200µg/200µl injection). Tumor size was assessed by digital caliper and luciferase expression measured weekly beginning w2. Mice were euthanized at experiment termination (5-6 weeks) and tumors, spleen, lungs, liver and brain collected. (B) Tumor growth curve with mean tumor volume ± SEM of 5 mice/treatment group showing αCD20-EndoP125A and Rituximab significantly inhibited tumor growth. (C) Representative images of spleen (D,F,H,J) Representative flow cytometry plots of indicated organs with percent of Jeko-1 GFP+ gated in green (E,G,I,K) Flow cytometry quantification of percentage of infiltrating Jeko-1 lymphoma cells within indicated organs. Results are mean percentage of Jeko-1 GFP+ cells within the live cell population ± SD of 4 indicated organs/treatment group. *Statistics were calculated and significant differences are indicated with p-values * < 0.05, ** < 0.01, *** < 0.001, **** < 0.0001.

Preparation of Jeko-1-Luc-GFP Cells

Jeko-1 cells were induced to express GFP/luciferase under the MSCV promoter using a lentiviral transduction system. For the lentiviral particle preparation, 293T cells were transfected with a combination of three plasmids: pCHD-MSCV-Luciferase-EF1-GFP, pMD2.G (Addgene #12259; VSV-G envelope expressing plasmid), and psPAX2 (Addgene #12260; 2nd generation lentiviral packaging plasmid). After one day media was exchanged, and then supernatant of the two following days was collected and concentrated 10 times by virus precipitation. Jeko-1 cells were then transduced with the virus containing supernatant at a multiplicity of infection of 10 in a media containing polybrene (8 µg/ml). Three days post transduction, GFP-positive cells were sorted and expanded.

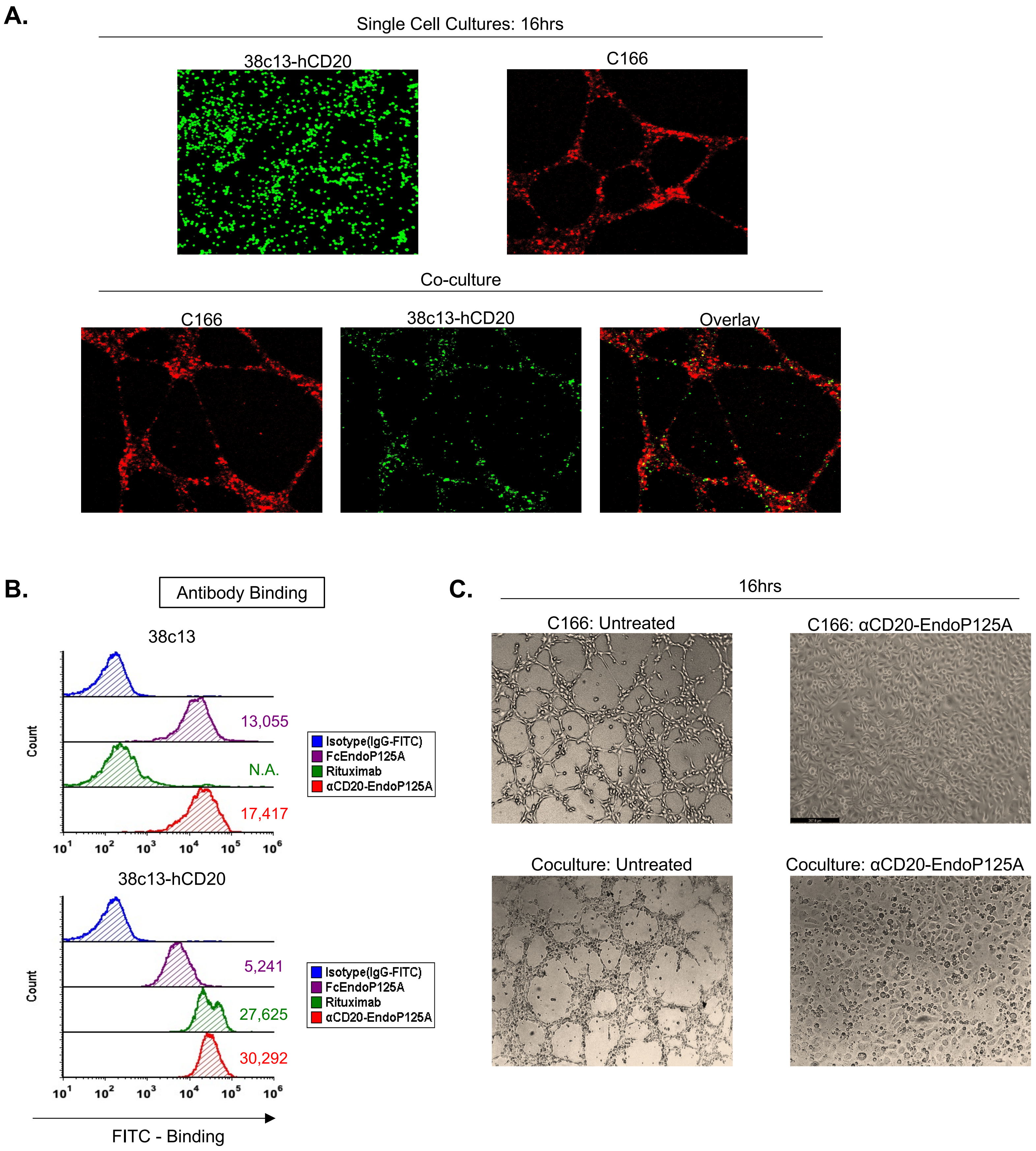

Supplementary Figure S10: 38c13-hCD20 aligns and associates with murine C166 CLS, antibody binding to 38c13-hCD20 cells, and αCD20-EndoP125A inhibition of murine CLS formation and alignment of 38c13-hCD20 - (A) To study murine lymphoma interaction with murine CLS, C166 (mouse endothelial cells, ATCC) were labelled with calcein red and 38c13-hCD20 with calcein green. Cells were either cultured alone or cultured in a 1:1 ratio on Matrigel. 38c13-hCD20 cells randomly disperse throughout Matrigel when cultured alone, but when co-cultured with the C166 which undergoes CLS formation they will migrate to and align with mouse CLS (B) Cells were blocked with mCD19/CD32 (BD Biosciences, cat#553142) then incubated with equimolar Fc-EndoP125A, Rituximab, or αCD20-EndoP125A. Samples were washed with PBS and incubated with αhuIgG-FITC (Sigma cat#F0132). Samples were then resuspended in staining buffer (1x PBS: 0.5%BSA, 2mM EDTA, 0.09%SA) and flow cytometry performed on an Attune NXT cytometer. Antibody binding to 38c13 and 38c13-hCD20 cells is displayed. Calculated MFI indicated (C) C166 were cultured alone or co-cultured with 38c13-hCD20 on Matrigel and either left untreated or treated with αCD20-EndoP125A. Inhibition of mouse CLS formation and alignment of 38c13-hCD20 cells was seen with αCD20-EndoP125A treatment

Table S3: Flow Cytometry Antibody List for Tissues Analysis of 38c13-hCD20 *in vivo* Experiment				
Target	Fluorescence	Antibody(µl)/Test	Manufacturer	Catalog#
Live/Dead Blue		0.5	Thermofisher	L34957
huCD20	FITC	20	BD Biosciences	555622
B220	eFluor 450	1	Invitrogen	48-0452-82
CD19	APC-Cy7	2.25	BioLegend	115530
CD45	BUV661	0.5	BD Biosciences	612975
CD4	BUV737	0.5	BD Biosciences	612761
CD3	BUV805	4	BD Biosciences	749276
CD8a	BV510	1	BioLegend	100752

A.

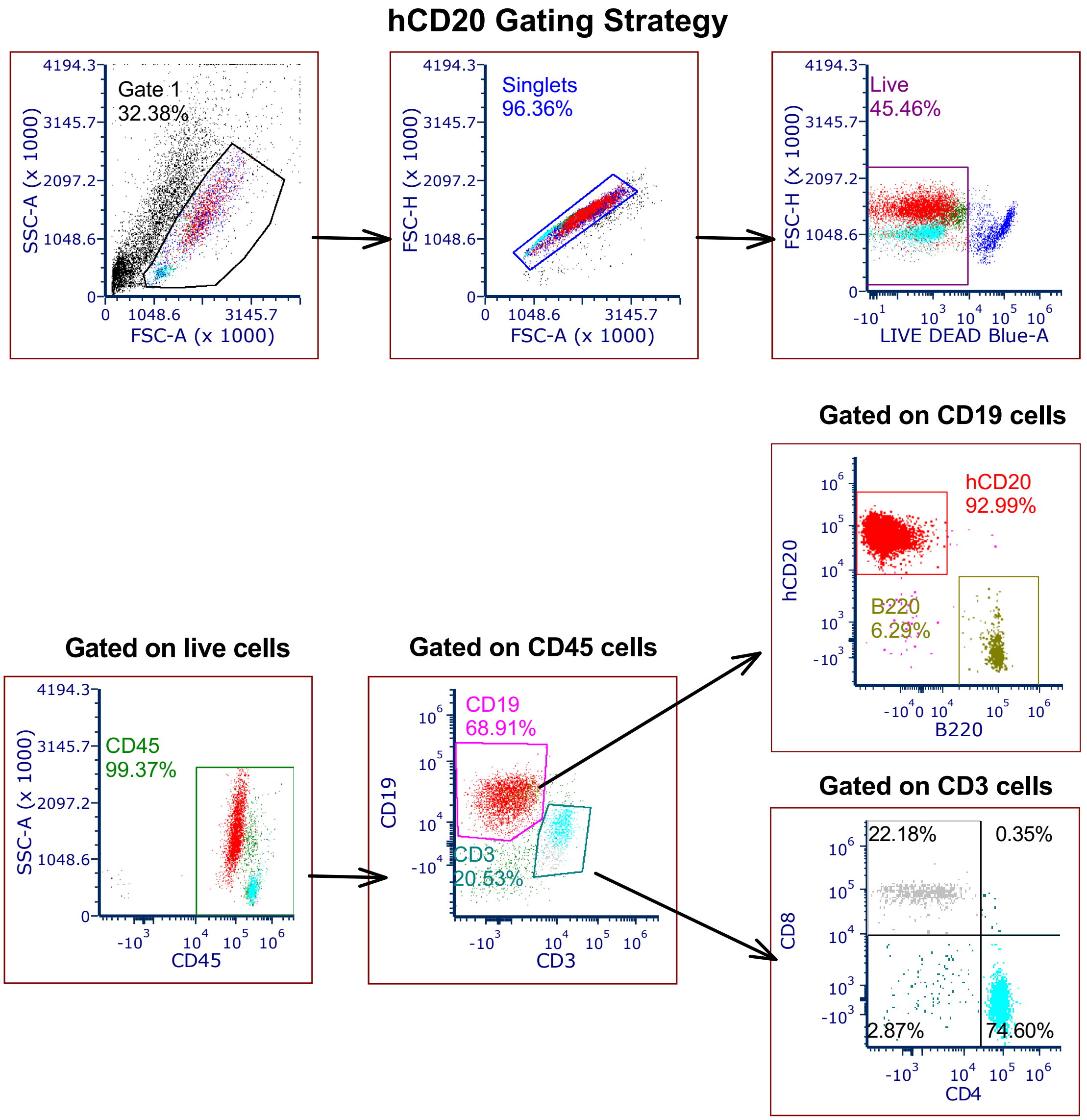

Supplementary Figure S11: hCD20 Gating- Tumors, lymph nodes, spleen, lungs, and brain were collected from naïve mice and tumor bearing mice from each of the treatment groups: PBS, Rituximab, Fc-EndoP125A and αCD20-EndoP125A. Tissues were processed as single cell suspensions. Cells were blocked with purified rat anti-mouse CD16/CD32 (BD Biosciences, cat#553142) then stained with antibodies (Antibody List above Supp Table 2) for 30 minutes at 4^0C in the dark. Samples were washed with PBS, resuspended in 500uL of staining buffer (1x PBS: 0.5%BSA, 2mM EDTA, 0.09%SA) and flow cytometry was performed on a Cytek Aurora Spectral Analyzer. (A) Flow cytometry gating Strategy used to distinguish lymphoma cells from normal mouse cells and determine amount of infiltrating 38c13-hCD20 lymphoma cells. Representative lymph node of PBS treated mice is displayed

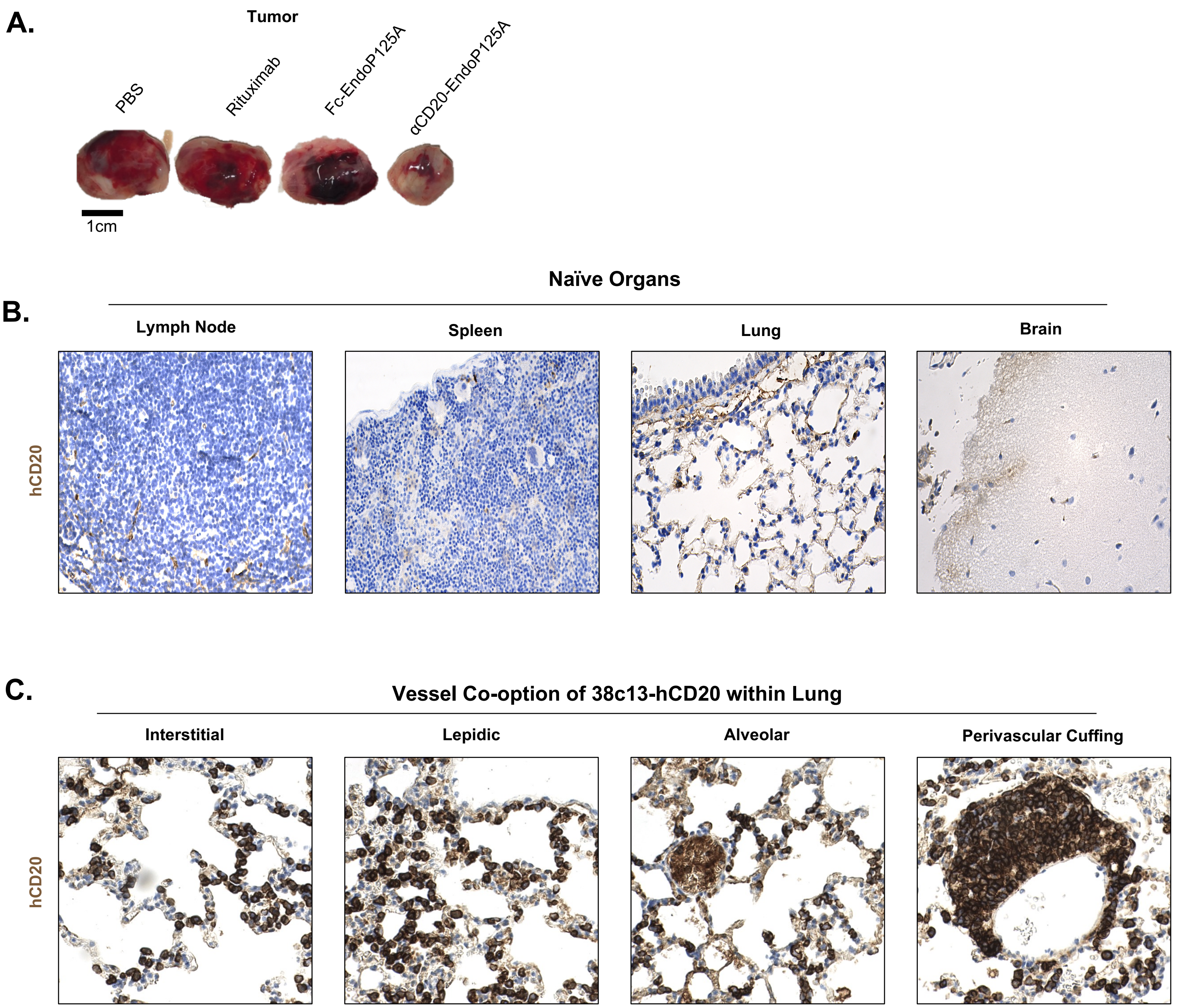

Supplementary Figure S12: Reduced vascularity of primary tumors by αCD20-EndoP125A and types of vessel co-option within the lung:(A)Representative images of tumors indicating decreased size and vascularity with αCD20-EndoP125A treatment (B) Lymph nodes, spleen, lungs, and brain were collected from naïve C3H mice unchallenged with 38c13-hCD20 tumors. Tissues were formalin fixed and paraffin embedded then sections were stained for hCD20. IHC displayed indicated negative hCD20 within various organs (C)Types of vessel co-option seen in lungs of 38c13-hCD20 tumor bearing mice treated with PBS, Rituximab or Fc-EndoP125A. $hCD20^{+}$ lymphoma cells stained brown. Both interstitial and lepidic growth patterns were seen in PBS, Rituximab and Fc-EndoP125A treated mice. In interstitial vessel co-option, tumor cells infiltrate lung interstitium and grow within the alveolar walls. In the lepidic pattern the tumor cells expand and begin to replace the pneumocytes which make up the alveolar wall. We also found several instances where tumor cells had grown in an alveolar or perivascular cuffing pattern in PBS and rituximab treated mice. In alveolar vessel co-option tumor cells expand to completely fill the alveolar space. In the perivascular cuffing pattern, the tumor cells co-opt large vessels within the lung and grow in cuff-like pattern of several tumor cells thick

References

1. Salcedo, R. & Oppenheim, J. J. Role of chemokines in angiogenesis: CXCL12/SDF-1 and CXCR4 interaction, a key regulator of endothelial cell responses. *Microcirculation* **10**, 359-370 (2003).
